# Impact of bacterial and fungal inoculants on the resident rhizosphere microbiome and the volatilome of tomato plants under leaf herbivory stress

**DOI:** 10.1093/femsec/fiad160

**Published:** 2024-02-08

**Authors:** Ana Shein Lee Díaz, Zhivko Minchev, Jos M Raaijmakers, María José Pozo, Paolina Garbeva

**Affiliations:** Department of Microbial Ecology, The Netherlands Institute of Ecology (NIOO-KNAW), Droevendaalsesteeg 10, 6708 PB Wageningen, the Netherlands; Department of Soil Microbiology and Symbiotic Systems , Estación Experimental del Zaidín, Consejo Superior de Investigaciones Cientfícias (CSIC), Calle Prof. Albareda, 1, 18008, Granada, Spain; Department of Microbial Ecology, The Netherlands Institute of Ecology (NIOO-KNAW), Droevendaalsesteeg 10, 6708 PB Wageningen, the Netherlands; Institute of Biology, Leiden University, Sylviusweg 72, 2333 BE, Leiden, the Netherlands; Department of Soil Microbiology and Symbiotic Systems , Estación Experimental del Zaidín, Consejo Superior de Investigaciones Cientfícias (CSIC), Calle Prof. Albareda, 1, 18008, Granada, Spain; Department of Microbial Ecology, The Netherlands Institute of Ecology (NIOO-KNAW), Droevendaalsesteeg 10, 6708 PB Wageningen, the Netherlands

**Keywords:** amplicon sequencing, insect herbivory, microbial consortia, microbial inoculation, rhizosphere communities, rhizosphere volatilome

## Abstract

Various studies have addressed the impact of microbial inoculants on the composition of the resident microbiome. How microbial inoculants impact plant metabolism and interact with the resident rhizobiota under herbivory stress remains elusive. Here, we investigated the impact of two bacterial and two fungal inoculants, inoculated as single species and as a synthetic community, on the rhizosphere microbiome and volatilome of tomato plants (*Solanum lycopersicum*) comparing nonstress conditions to exposed to leaf herbivory by *Spodoptera exigua*. Based on amplicon sequencing analysis, rhizobacterial community composition was significantly affected by all four inoculants and the magnitude of this effect was dependent on herbivory stress. Fungal community composition was altered by the microbial inoculants but independent of herbivory stress. The rhizosphere volatilome was impacted by the microbial inoculation and differences between treatments were evened under herbivory stress. Each microbial inoculant caused unique changes in the volatilome of stressed plants but also shared similar responses, in particular the enhanced production of dimethyl disulfide and benzothiazole. In conclusion, the introduction of microbial inoculants in the tomato rhizosphere caused unique as well as common changes in the rhizosphere microbiome and volatilome, but these changes were minor compared to the microbiome changes induced by herbivory stress.

## Teaser

Changes in rhizospheric bacterial and fungal communities and volatilome of tomato plants under microbial inoculation of beneficial microbes as single species and as a synthetic community under leaf herbivory stress conditions.

## Introduction

In the current agricultural scheme, there is a great need for sustainable alternatives to chemical pesticides and fertilizers. In this context, plant-beneficial microbes are considered an environmentally friendly alternative for improving plant health and defense (Finkel et al. 2017). Microbial inoculants typically contain a strain of specific plant-associated beneficial bacterial or fungal species. Inoculation consists of introducing the microbes into the soil or onto planting materials, for example, by seed coating, root dipping, or soil drenching. However, the potential use of microbial inoculants is still questioned due to their variable efficacy under field conditions. Also, the mechanisms underlying their ecological interactions with soil microbes and the host plant are largely unknown. Particularly for the root- or soil-inoculated microbes, successful rhizosphere colonization is crucial for the establishment of a beneficial relationship with the plant (Romano et al. 2020). To achieve a sufficient colonization level, different strategies are often followed to improve microbial survival and adaptation to the prevailing natural conditions. These include the introduction of high numbers of bacterial or fungal cells (often higher than 10^7^ cells/g), elaborating on specific formulations, or repeated applications (Kaminsky et al. 2019). Another approach is to apply beneficial microbes as a combination of different species (known as microbial consortia or synthetic communities). This is considered more advantageous than the application of single strains due to potential synergies in their effects on plant protection or microbial persistence in the environment (Bradáčová et al. 2019b). Another factor influencing the success of microbial colonization is the species richness and evenness of the resident soil microbial community. It has been observed that resident microbial communities with higher diversity can better resist the alterations in community function and structure due to microbial invasions (Mallon et al. 2015) and that despite the outcome of the colonization, a legacy effect remains detectable in the niche structure of the community (van Elsas et al. 2012).

To improve the efficacy of microbial inoculants, research has focused on understanding the ecological interactions between inoculated microbes and the recipient ecosystem. Variable results have been reported in different studies regarding the impact of inoculants on resident soil microbial communities, depending on the studied system, the sampling procedure, and the detection method used (e.g. culture-dependent, microscopy, or metagenomic) (Romano et al. 2020). Although there is little consensus about the degree of such effects (Trabelsi and Mhamdi 2013), a recent meta-analysis reported that out of 108 studies on the impact of inoculation on resident microbial communities, 86% altered the soil communities either on the short or long term (Mawarda et al. 2020). The majority of the reported studies assessed the impact of microbial inoculation on plant growth, biocontrol of plant pathogens, or plant tolerance to abiotic stresses such as drought or soil contamination (Mawarda et al. 2020). However, the studies about the impact of diverse microbial inoculants are still biased toward single bacterial strains: out of the 108 studies, 86 assessed the impact of single bacterial inoculants, 22 of fungal inoculants, and only two studies of microbial consortia (Mawarda et al. 2020). Fewer studies focused on microbial-induced systemic resistance (ISR), especially regarding resistance to insect pests. ISR-triggering microbes induced physiological and metabolic changes in the plant that result in an enhanced defense status against both biotic and abiotic stresses (Pieterse et al. 2014, Jeon et al. 2022). Microbial inoculation can impact the natural communities not only through microbe–microbe interactions such as niche and nutrient competition, antibiosis, or priority effects (Fukami 2015) but also via altering the plant's physiology, and therefore plant’s ecological interaction with the rhizosphere microbiota.

The plant’s interaction with microbial inoculants is context-dependent, and the outcome of the mutualistic interaction can vary depending on the plant’s physiological status (Lee Díaz et al. 2021). Aboveground organisms, such as insect pests, can impact belowground plant defense (alteration of root exudates and defense compounds) and the soil community composition (Bezemer and van Dam 2005). Under foliar insect herbivory stress, plants were reported to interact with the rhizosphere microbiota and recruit microbes that can help alleviate or fight the stressor (Yi et al. 2011). Several studies have shown that both aboveground and belowground herbivory stress can induce changes in the rhizosphere microbial communities (Hu et al. 2018, Pineda et al. 2020). The subsequent recruitment and assembly of the rhizosphere are largely driven by metabolic changes in root exudates and volatile compounds (Rizaludin et al. 2021). However, how microbial inoculation can impact plant chemistry and concomitantly community assembly under stress has received less attention. Plants’ volatile emissions can vary according to their physiological status; e.g. stress or flowering, and aboveground stresses can have an impact on the root compartment. This has been shown in studies where leaf herbivore-stressed plants presented a different root volatilome than nonstressed plants (Danner et al. 2015, Lee Díaz et al. 2022). Microbial inoculants and soil microbes can also emit volatiles in response to environmental factors such as plant hormones, nutrient resources, and microbe–microbe interactions (Schulz-Bohm et al. 2015). Assessing the impact of microbial inoculation not only from a community composition perspective but also from a metabolomic perspective will provide a more comprehensive understanding of the untargeted inoculation effects (Mawarda et al. 2020).

In this study, we investigated the impact of five microbial inoculant treatments on the resident rhizosphere microbial communities and the volatilome of tomato (*Solanum lycopersicum*) plants under leaf herbivory stress. We inoculated tomato plants with each of two phylogenetically diverse bacteria species; *Bacillus amyloliquefaciens* (Ba) and *Pseudomonas azotoformans* (Pa), and two fungal species; *Trichoderma harzianum* (Th) and *Rhizophagus irregularis* (Ri) to investigate common or inoculant-specific patterns on their impact on the resident microbial communities and the root volatilome. In addition to being inoculated alone as single species, the microbes were inoculated as a consortium or synthetic community (SynCom) to test whether the combination affects the natural communities differently than single strains. To compare the inoculation effect under stress, a set of the inoculated plants was subjected to continuous leaf herbivory stress for 15 days by the chewing lepidopteran insect *Spodoptera exigua*. Additionally, we studied the root volatilome under differential control and stress conditions to assess changes in root chemistry during the first 24 h of the herbivory attack. Our study revealed that the different microbial inoculants impacted the rhizosphere communities in terms of diversity and structure and also in terms of rhizosphere volatilome in a predominantly unique manner.

## Materials and methods

### Microbial strains and preparation of the inoculants

Microbial strains were provided by Koppert (Berkel en Rodenrijs, the Netherlands); the bacterial strains were (Ba) *B. amyloliquefaciens* strain CECT 8238 and (Pa) *P. azotoformans* F30A, while the fungal strains were (Th) *T. harzianum* T22 and the arbuscular mycorrhizal fungi *R. irregularis* (Ri) MUCL 57021. All inoculants were prepared according to Minchev et al. (2021). *Bacillus amyloliquefaciens* was grown in tryptone soya agar (TSA) at 28ºC for 24 h. For spore production, liquid difco sporulation medium was prepared as described by Nicholson and Setlow (1990) and inoculated with a single colony from TSA culture and incubated for 48 h at 28ºC with rotatory shacking at 200 r/m. The resulting spores were separated from the liquid medium by centrifuging for 15 min at 5000 r/m and resuspended in sterile tap water at a final concentration of 1 ×10^7^ spores/ml. *Pseudomonas azotoformans* was grown on TSA at 28ºC for 24 h. Next, a liquid preculture was prepared with tryptone soya broth (TSB) inoculated with a single colony from TSA culture and incubated overnight at 28ºC with rotatory shaking at 200 r/m. Then 1 ml of preculture was used to inoculate 25 ml of TSB and incubated at 28ºC with rotatory shaking for 3 h when the bacterial growth is in the exponential phase. Bacterial cell concentration was quantified by measuring the optical density (OD) at 620 nm, then the cells were separated from the medium (Table S1, Supporting Information) by centrifuging for 15 min at 5000 r/m and resuspended in tap water at a final concentration of 1 ×10^7^ cfu/ml. *Trichoderma harzianum* was grown on potato dextrose agar at 25ºC for 7 days. Spores were recovered from the sporulated culture in sterile tap water, spore concentration was quantified using a Neubauer hemocytometer and adjusted to a final concentration of 1 × 10^7^ spores/ml. *Rhizophagus irregularis* was grown in monoaxenic culture on minimal (M) medium (Bécard and Fortin 1988) with *Agrobacterium rhizogenes* transformed carrot roots as host (St-Arnaud et al. 1996). Spores were extracted from sporulated culture and resuspended in tap water to a final concentration of 1000 spores/ml. Finally, a synthetic microbial consortia (SynCom) inoculum was prepared mixing microbial suspensions of 1 ×10^7^ spores/ml of *B. amyloliquefaciens, P. azotoformans, T. harzianum*, and 1000 spores/ml of *R. irregularis*.

### Soil preparation, seedling inoculation, and plant growing conditions

The soil was obtained from an olive tree field in Gójar with annual organic manure fertilization under the trees (Granada, Spain) (37°05′49.5“N; 3°36′19.1″W; 810 m elevation). The soil used was a calcareous cambisol obtained by digging at a depth of 25 cm in the inter-rows between trees. Collected soil was sieved through a 1.5-mm pore-diameter and tyndalized in three consecutive days at 95°C for 45 min (Tyndall 1877). The growing substrate consisted of a mixture of tyndalized soil and sterile sand in proportion 1:1 v/v. Finally, 300 ml pots were filled with the growing substrate.

Seed surface sterilization, germination, and microbial inoculation were done according to Minchev et al. (2021). Briefly, tomato *Solanum lycopersicum* cv Moneymaker seeds obtained from Vreeken’s Zaden (Dordrecht, the Netherlands), were surface sterilized by immersion in 5% sodium hypochlorite for 10 min, rinsed with sterile water three times for 10 min and incubated in sterile vermiculite for 7 days at 24ºC. Seedlings were root inoculated by pipetting 1 ml of a microbial suspension of each microbe and the consortium during transplantation. A total of 72 plants were grown: 12 replicates were inoculated per microbial treatment with the corresponding microbial solution (Ba = *B. amyloliquefaciens*, Pa = *P. azofotormans*, Th = *T. harzianum*, Ri = *R. irregularis*, and SynCom = synthetic community). Similarly, 12 control plants were mock-inoculated with sterile tap water. All plants were grown under same conditions for 6 weeks in greenhouses at 24–20°C (day–night) and relative humidity of 60%. Plants were watered twice per week with water and once per week with Long Ashton nutrient solution (Hewitt 1966) with reduced phosphate concentration (50%) to facilitate mycorrhizal establishment. After the completion of 6 weeks of growth, half of the plants per treatment were subjected to herbivory stress for another 2 weeks (further details in herbivory stress induction section), therefore final harvest was carried out at week 8 of growth for shoot biomass, shoot nutrient content, rhizosphere communities and microbial colonization.

### Herbivory stress induction

*Spodoptera exigua* (Lepidoptera, Noctunidae) eggs provided by Entocare (Wageningen, the Netherlands), were reared after hatching at room temperature (∼21°C) on an artificial diet (Table S1, Supporting Information). For each microbial treatment, six out of the 12 microbial-inoculated 6-week-old tomato plant plants were subjected to herbivory by placing third-instar larvae in a clip cage (stressed plants; H+), while an empty clip-cage was placed in the remaining six nonstressed (H−) control plants per treatment. Two caterpillars were placed inside a clip cage on the apical leaflet of the third true leaf and moved to another leaflet of the same leaf before the leaflet was fully consumed. Caterpillars were checked every 48 h and dead caterpillars were replaced to continue the herbivory stress for 2 weeks until the harvesting of the plants (at final stage of 8 weeks).

### Micorrhizal colonization detection

Mycorrhizal colonization in Ri, SynCom and noninoculated plants was quantified histochemically. Upon harvesting, roots were washed, cut into pieces of 1 cm and cleared by incubating them in 10% potassium hydroxide at 4ºC for 2 days. After clearing, root samples were rinsed three times with deionized water, acidified with 2% acetic acid for 5 min at room temperature and stained by immersion in a solution of 5% ink (Lamy, Germany) and 2% acetic acid for at least 30 min at room temperature. The excess of ink solution was removed by rinsing the root samples three times with deionized water (García et al. 2020). Mycorrhizal colonization was evaluated by quantifying the percentage of root length colonized by the fungus according to the grid line intersection method (Giovannetti and Mosse 1980) under stereo microscope Motic SMZ. Briefly, around 200 root pieces were randomly placed in a Petri dish with grid of lines of 1 cm and mycorrhizal colonization was assessed by counting positive (mycorrhizal) and negative (nonmycorrhizal) roots/gridline intersections, counting between 100 and 200 intersections. Finally, the percentage of root length colonized by the fungus was calculated by dividing the number of positive intersections by the total number of intersections and multiplying by 100 (García et al. 2020).

### Plant nutrient content analysis

Leaf material was oven dried at 60°C and separated from stems and branches manually. Leaves were crushed in a mortar and further ground in the Tissue Lyser II (Qiagen, Germany) inside a 2-ml Eppendorf tube with metal beads at a maximum speed for 3 min. An average of 2.2 mg aliquot was weighed per sample and folded in tin cups for analysis of carbon (C) and nitrogen (N) content in the Flash 1112 Elemental Analyzer (Thermo Scientific, MA, USA). C, N, and C/N ratio data were provided in µg and normalized according to sample weight (in mg).

### Rhizosphere soil DNA extraction and sequencing

Rhizosphere soil was collected from root-attached soil of 8-week-old plants. Sterile vials of 15 ml were filled with soil adhering around fine roots from the bottom of the pot. Soil samples were frozen in liquid nitrogen and stored at −80°C until further analysis. An aliquot of ∼806 ± 0.1 mg of each soil sample was used for DNA extraction and fine roots were removed manually with sterile utensils. Rhizospheric DNA extraction was done according to the manufacturer’s instructions with the DNeasy® PowerSoil® Pro Kit (Qiagen, Hilden, Germany). DNA quantity and quality were measured with NanoDrop® and Qubit® fluorometers. DNA samples were sequenced using the Illumina NovaSeq6000 or MiSeq system and demultiplexed by BaseClear N.V. (Leiden, the Netherlands). For bacteria, the V3–V4 region of the ribosomal (rRNA) 16S gene was amplified with the 341F-NXT/785R-NXT primers. For fungi, internal transcribed spacer (ITS) in the ribosomal (rRNA) gene was amplified with the ITS86F/ITS4 primers according to Beeck et al. (2014). Sequencing yielded an average of 33 416 bp read pairs for fungal sequences and 38 369 bp for bacterial sequences. The average quality was 34.7 and 34.3 for fungal and bacterial sequences, respectively.

Raw FASTQ read sequence files were generated with blc2fastq (v2.20, Illumina), and quality assessment was performed with the FASTQC quality control tool (v0.11.8). Raw data sequences were analyzed with RStudio version 2022.07.2+576. DADA2 pipeline (1.16) was used for trimming, primer removal, filtering, error calculation, assembly of ASVs, and taxonomic classification to obtain the ASV abundance and ASV taxonomy tables (Callahan et al. 2016). The following R packages were used; *dada2* (v1.18.0) (Callahan et al. 2016: 2), *ShortRead* (v1.48), *Biostrings* (v2.58), and *tidyverse*. The function *cutadapt* (v 3.4) was used to trim the sequences where 280 bp and 250 bp were truncated for bacterial 16S samples and 200 bp and 180 bp were truncated for fungal ITS sequences from forward and reverse sequences, respectively. Chimaeras were removed and taxonomy was assigned using the Silva v1.38.1 database (Quast et al. 2013) for bacteria and Unite v8.3 database (Nilsson et al. 2019) for fungi.

### Volatile trapping and data analysis

Rhizosphere volatiles were trapped through passive diffusion by placing one Tenax® (Markes International, Llantrisant, United Kingdom) trap containing 200 mg Tenax/tube type TA 60/80trap inside a metal holder in each pot, reaching an approximate depth of 5 cm. Metal holders were stainless steel cylinders with perforations allowing the air entrance while protecting the trap from soil contamination. Tenax traps were placed before the onset of the herbivory stress for 24 h (T0). Upon the leaf-herbivory stress induced by *S. exigua*, new Tenax traps were placed in the same pots where T0 was measured. Traps were placed 24 h after the onset of the herbivory stress for a 24 h period, thus collecting the volatiles during the initial 24–48 h herbivory stress induction period (T1). Traps were tightly closed and stored at room temperature until measurement. Before utilization, Tenax traps were preconditioned by heating at 300°C for 45 min under helium flow (5 l/min).

Full details of Tenax measurement regarding thermal desorption, GC/Q-TOF measuring conditions, calibration, data analysis, and compound identification are described by Lee Díaz et al. (2022). Briefly, volatiles was thermo-desorbed from Tenax traps at 240°C for 8 min and then transferred to an ultra-inter column (122–5532 UI, Agilent Technologies, Inc., Santa Clara, CA, USA) of the GC/Q-TOF (model Agilent 7890B GC and the Agilent 7200A Q-TOF). An n-alkane (C8–C20) standard solution was spiked at the beginning of the run for calibration. Mass spectra of compounds were acquired in full-scan-mode and GC/Q-TOF raw data was translated to .cdf format and analyzed with MzMine v2.53 (Pluskal et al. 2010, 2020) for mass feature detection and peak intensity quantification. Parameters used for GC-MS peak intensity tables (Du et al. 2020) are available in Table S3 (Supporting Information). Peak intensity tables were used in combination with chromatogram data (Mass Hunter Qualitative v10, Agilent Technologies) for manual identification of volatile compounds comparing the mass spectrum of target compounds with the NIST 2020 database (V2.20, National Institute of Standards and Technology, USA).

### Statistical analysis

Statistical analysis on nutrient data was done with RStudio version 2022.07.2+576. 2-way ANOVA analysis with sums of squares type III was applied to C, N, and CN ratio relative values for differences between microbial treatments and herbivory stress. *Post hoc* analysis was applied with the Tukey Honest Significant Difference (HSD) test. Interaction figures elaborated with the *emmeans* package from a linear model test for interacting factors.

Statistical analysis of amplicon sequence data of ASV abundance and taxonomy tables were statistically analyzed with the *phyloseq* (McMurdie and Holmes 2013) and *metagenomeSeq* (Paulson et al. 2013) R packages for relative abundance, alpha, and beta diversity, and ASV contrast analysis of bacterial and fungal communities. Alpha diversity indexes Shannon, Chao1, Observed, and Inverse Simpson (InvSimpson) were calculated with the *estimate_richness* function of the *phyloseq* package. Statistical analyses of diversity indexes were done applying a two-way ANOVA test with microbial treatment (with six levels for Control, Ba, Pa, Th, Ri, and SynCom) and herbivory stress (with two levels for nonherbivory control and herbivory-stressed) as factors. *Post hoc* analysis was performed with Tukey-HSD. Kruskal–Wallis statistical test was applied when data were not normally distributed. Beta diversity was calculated over normalized ASV counts with the cumulative sum scaling (CSS) *cumNorm* function of the *phyloseq* package. The distance matrix was built according to the Bray–Curtis method, and Permutational multivariate analysis of variance (PERMANOVA) analysis was done with the *adonis* function of the *vegan* package and the *post doc* test with the *pairwise.adonis* package at 9999 permutations. Contrast analysis of ASV differential abundance was done with the *metagenomeSeq* package. CSS-normalized samples were filtered for estimated effective samples and fitted for differential testing with the *fitZig* function. Contrast matrixes were compared pairwise to obtain ASVs with a logarithmic FC statistically significant (*P*-value < .05). ASV differential analysis representations were elaborated with Fluorish Studio (https://flourish.studio/).

Statistical analysis of peak intensity tables of volatile data was done with MetaboAnalyst v.5.0 (Pang et al. 2021). Peak intensity tables were analyzed with two different statistical tests; one to test the effect of the microbial inoculation on the volatilome of nonstressed or stressed plants (analysis one), and a second analysis to test the effect of the stress within each microbial treatment (analysis two). For analysis one, normalization was done by log10 transformation and Pareto-scaling. Statistical differences between treatments were analyzed with one-way ANOVA for timepoint T0 (nonstressed) and in a separate analysis for T1 (stressed). *Post hoc* analysis of Tukey’s HSD was performed for pairwise comparisons between treatments of significantly different compounds. Data was represented with a hierarchical clustering as a heatmap. Treatment group averages were clustered according to Euclidean distance similarity measures according to Ward’s linkage algorithm. For analysis two, normalization was done by log10 transform and autoscaling. Statistical differences within one treatment before and after herbivory were analyzed with a paired Fold Change (FC) analysis. Paired (nonindependent data) FC analysis counts the total number of FCs above a threshold of 2 and values are provided in the log2 scale. FC representation with bar plots was elaborated with R Studio version 2022.02.3 build 492.

## Results

### Microbial colonization, plant biomass, and nutrient content

The presence of the microbial inoculants in the rhizosphere at 8 weeks after inoculation was confirmed by RT-qPCR, for all treatments (data not shown). In addition, root colonization by the introduced arbuscular mycorrhizal fungus *R. irregularis* was confirmed by histochemical staining of fungal structures within the roots. The root mycorrhization percentage ranged from 20% to 50%, and differences between treatments were statistically significant. (Figure S1, Supporting Information). Under herbivory stress, Ri-inoculated plants had a significantly higher mycorrhization percentage than nonstressed control plants (*P*-adj = 0.006) and SynCom-inoculated plants (*P*-adj = 0.031). This result suggests a different plant–AMF interaction when *R. irregularis* is inoculated as single species (Ri) or in a SynCom under leaf herbivory stress.

**Figure 1.**
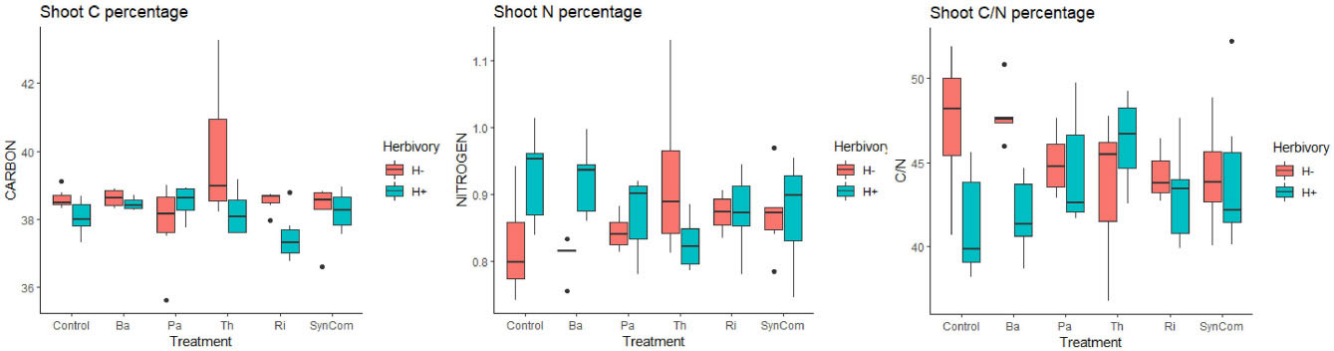
Impact of microbial inoculation and leaf herbivory stress on shoot C, N, and C/N content. Boxplots of relative shoot nutrient content (µg/mg) of 8-week old tomato plants inoculated with microbial treatments Control (mock-inoculated), Ba, Pa, Th, Ri, and SynCom. Red color indicates plants without herbivory (H−), while plants subjected to herbivory stress over 2 weeks are indicated in blue (H+).

According to two-way ANOVA, microbial inoculation did not have an effect on plant shoot biomass. The herbivory stress led to a slight reduction on the shoot biomass of Ri-inoculated plants (Ri H+ vs. H−; *P*-adj = 0.015) (Figure S2, Supporting Information). The effect microbial inoculation on the relative shoot nutrient content (µg/mg), was herbivory-stress dependent, since two-way ANOVA showed that the interaction between the inoculant treatment and the herbivory significantly impacted C, N, and C/N ratio (Treatment*Herbivory; C *P*-value = .02, N *P*-value = .002, and C/N *P*-value = .007) (Fig. 1). The herbivory stress altered the relative content of shoot C and C/N ratio, and a significant interaction between microbial treatment and herbivory stress was observed for all nutrient parameters (C, N, and C/N) (Table S4, Supporting Information). Control- and Ba-inoculated plants presented a similar trend in N and C/N ratio in response to herbivory; for both treatments, N significantly increased upon herbivory (and thereby a reduction of C/N ratio) (Table S4, Supporting Information). Under herbivory stress, Th-inoculated plants showed opposite, yet not statistically significant, nutrient responses for N and C/N ratio compared to Control and Ba-inoculated plants (Fig. 1). Interestingly, the nutrient content in SynCom-inoculated plants was barely altered by herbivory stress (Fig. 1).

### Effect of microbial inoculants on the composition of rhizosphere resident microbial communities

#### Bacterial communities

**Figure 2.**
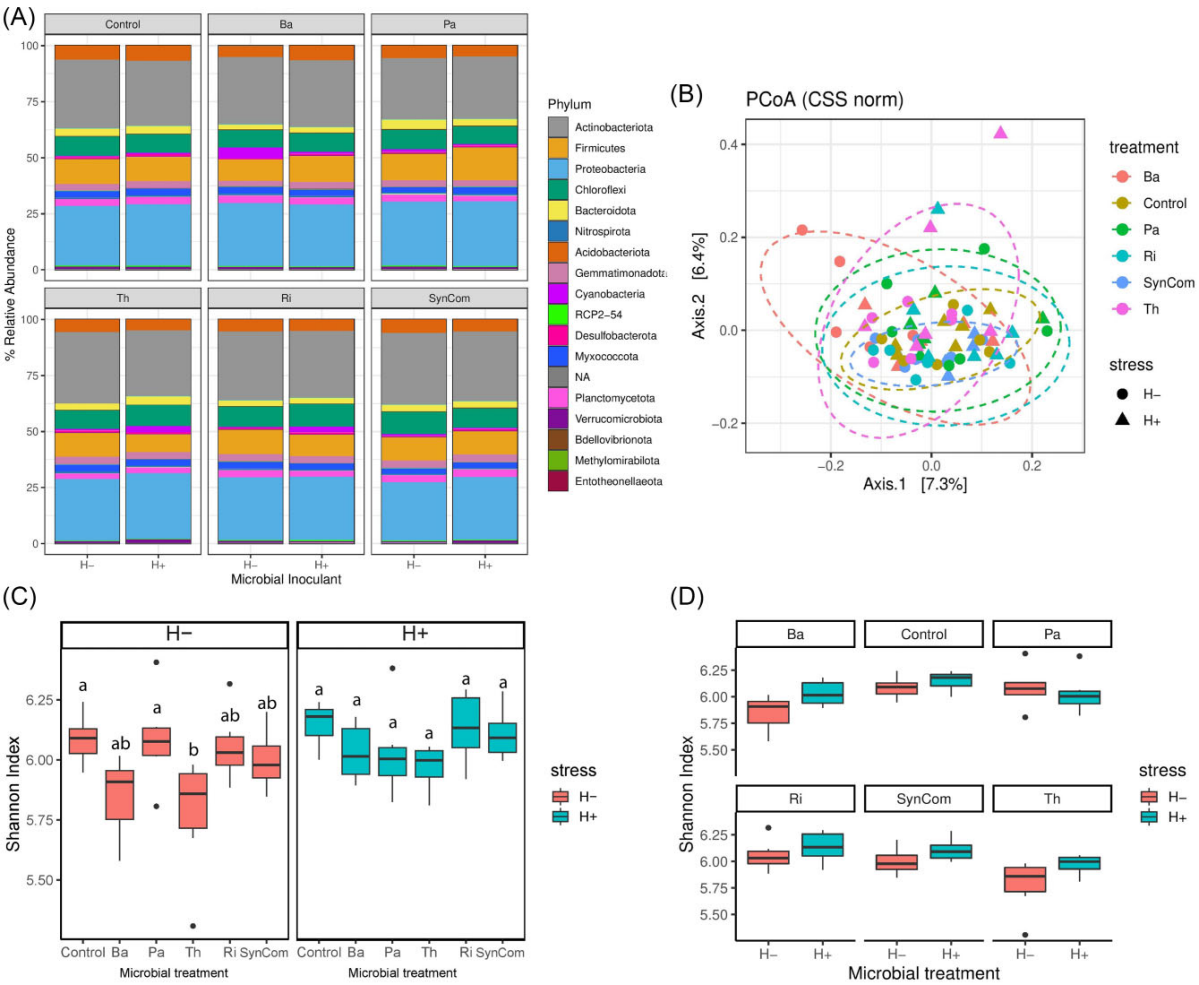
Impact of microbial inoculation and herbivory on bacterial communities and diversity. Relative abundance (A), beta diversity (B), and alpha diversity (C) and (D) of tomato rhizobacterial communities per microbial treatment; noninoculated (Control), *B. amyloliquefaciens* (Ba), *P. azotoformans* (Pa), *T. harzianum* T22 (Th), *R. irregularis* (Ri), and the SynCom, with (H+) and without herbivory (H−) stress. (A) Bar plot of bacterial phyla relative abundance grouped by microbial inoculant treatment. Each stacked bar represents the average relative abundance of the different taxa (color guide on the legend) according to the herbivory stress (the left bar for H−; nonstressed, the right bar for H+; herbivory-stressed). (B) Principal Coordinates Analysis (PCoA) plot of Euclidean distances representing community dissimilarities between treatments (color guide) and stress (circles for nonstressed, and triangles for stressed plants). The *x*- and *y*-axis represent the components explaining the maximum variability between samples. Box plots of alpha diversity Shannon index for comparisons among microbial treatments within one stress condition (C) or within a microbial treatment (D). Statistical differences between comparisons (*P* < .05) are noted with different letters (C).

Resident rhizobacterial communities presented a total of 7598 unique ASVs encompassing 35 phyla and 534 genera. The community composition was similar across microbial inoculant treatments and herbivory stress. The top 10 most abundant phyla were Actinobacteria (30%), Proteobacteria (28%), Firmicutes (11%), Chloroflexi (9%), Acidobacteria (6%), Gemmatimonadota (3%), Planctomycota (3%), Bacterioidota (3%), Myxococcota (3%), and Cyanobacteria (1%) (Fig. 2A). Of the 534 bacterial genera identified, only 35 presented a relative abundance higher than 0.5%. The top 10 most abundant genera were *Microvirga* (6.4%), *Bacillus* (6%), *Rubrobacter* (5.5%), *Pseudarthrobacter* (3.7%), *Skermanella* (3.3%), *Nocardioides* (2.8%), *Marmoricola* (2.4%), *Rubellimicrobium* (2%), *Bryobacter* (2%), and *Sphingomonas* (1.7%). Despite similar relative abundance profiles at the phylum level across microbial inoculant treatments and herbivory stress, the resident rhizobacterial communities were significantly impacted by the microbial inoculants, but not by the herbivory stress (Table 1). *Post hoc* pairwise tests showed that all microbial inoculant treatments, except *P. azotoformans* (Pa), significantly altered the composition of the resident rhizobacterial communities compared to noninoculated Control plants (Table 1). Also, SynCom-inoculated plants significantly differed from Ba- and Th-inoculated plants, showing a different effect of these two microbes when inoculated as a single strain than as a consortium. Although the herbivory stress did not have an overall significant effect on the composition of the rhizobacterial communities, PERMANOVA analysis performed within each microbial treatment showed that herbivory stress significantly altered communities only in Ba-inoculated plants (Table 1).

**Table 1.** Bacterial beta diversity. PERMANOVA test values for the effect of microbial inoculation and stress on bacterial communities. Pairwise test values for differences between bacterial communities.

Bacterial communities			
PERMANOVA	F model	*R* ^2^	Pr(> F)
Microbial treatment	1.379	0.0945	0.001
Herbivory stress	1.475	0.0165	0.127
**Pairwise** **^1^**	**F model**	***P*-value**	***P*-adjusted**
**Between treatments**			
Control vs. Th	1.730	.0001	0.002
Control vs. SynCom	1.635	.0003	0.002
Control vs. Ba	1.737	.0018	0.007
Control vs. Ri	1.454	.0063	0.018
SynCom vs. Th	1.465	.0018	0.007
SynCom vs. Ba	1.502	.007	0.018
**Within treatment**			
Ba H+ vs. H−	1.374	.0446	0.0446

1 Pairwise Adonis values for comparisons between treatments (only statistically significant comparisons reported for Control and SynCom). Pairwise comparison test values within treatment for comparisons of one microbial treatment between two stress conditions (nonstressed; H−, herbivory-stressed; H+). Only significant comparisons are reported.

The multivariate principal coordinates analysis (PCoA) showed absence of clustering of treatments, with an overlap between stressed and nonstressed communities (Fig. 2B). Two-way ANOVA analysis showed that bacterial alpha diversity was significantly impacted by microbial inoculation (F-value = 4.25, *P* = .002) and herbivory stress (F-value = 6.31, *P* = .015). The microbial inoculation effect was statistically significant only in nonstressed plants, where Control and Pa-inoculated plants had a higher Shannon index than Th-inoculated plants (Fig. 2C). The herbivory stress caused an overall significant increase in alpha diversity, but no significant differences among treatments were observed (Fig. 2C). Despite the herbivory stress having a general effect on diversity, only Pa-inoculated plants showed a reduction of the Shannon index under stress (Fig. 2D). An additional description of rhizobacterial alpha diversity values (Chao1, Observed, and Inverse Simpson indexes) and statistical analysis performed on pairwise differences between treatments under different stress levels are provided in Table S5 (Supporting Information).

Contrast analysis of ASV’s relative abundances confirmed that Ba- and Th-inoculated plants had the largest number of significantly reduced ASVs compared to noninoculated Control plants. In absence of herbivory, 39 out of the 48 differentially abundant ASVs between Control and Ba-inoculated plants were significantly reduced in control plants (Table 2). The Th was the second inoculant with the most differential ASVs, where 19 ASVs were reduced upon inoculation (Table 2). Similarly, under stress, Ba and Th treatments also accounted for the largest number of differential ASVs (50 and 56, respectively). The remaining treatments presented a higher number of differential ASVs in comparison with the Control under herbivory stress: 36 for SynCom-, 34 for Ri-, and 35 for Pa-inoculated plants (Table 2). Overall, the number of enriched ASVs relative to the Control was higher under herbivory stress.

**Table 2.** Bacterial ASVs differential relative abundance. Total numbers of significantly reduced or enriched (fold-changed) ASVs under nonherbivory (H−) or leaf-herbivory stress (H+) in control plants with respect to the compared microbial treatment.

	Nonherbivory (H−)			Herbivory (H+)		
Comparison	Reduced	Enriched	Total	Reduced	Enriched	Total
Control vs. Ba	39	9	48	25	25	50
Control vs. Th	19	7	26	33	23	56
Control vs. SynCom	6	7	13	12	24	36
Control vs. Ri	3	3	6	14	20	34
Control vs. Pa	2	1	3	14	21	35

In the absence of herbivory stress, there were no commonly reduced ASVs relative to the Control among all microbial treatments. Only Ba-inoculated and Th-inoculated plants shared the largest FC reduction of ASV130 (*Cellulomonas*, Actinobacteriota) and the unknown ASV455. In Ba-inoculated plants, the most reduced bacterial orders were Burkholderiales (Proteobacteriota), Gemmatales (Planctomycetota), Gemmatimonadales (Gemmatimonadota), Geobacterales (Desulfobacterota), Polyangiales (Myxococcota), and Streptosporangiales (Actinobacteriota). On the other hand, there were more commonly enriched ASVs in microbial treatments compared to noninoculated Control plants. The ASV363 *Ramilibacter* (Proteobacteria) was commonly enriched in all treatments compared to Control. The treatments which shared the most enriched ASVs were Ba-, Th-, and SynCom-inoculated plants, sharing five commonly enriched ASVs; ASV260, ASV482, and ASV594 identified as *Blastococcus* (Actinobacteriota), ASV411 (Actinobacteriota), and ASV535 *Ohtaekwangia* (Bacteroidota) (Figure S3, Supporting Information). Under herbivory stress, a similar pattern was observed as in nonstress; Ba- and Th-inoculated plants presented the highest number of reduced ASVs compared to controls. Both treatments commonly shared eight reduced ASVs, with the largest FC reduction in ASV494 (Actinobateriota) (Figure S4, Supporting Information). Similarly, to nonherbivory, more enriched ASVs were found in common among treatments. All four microbial treatments presented in common the largest enrichment of the same genus, ASV66 *Geodermatophilus* (Actinobacteriota), as well as other six yet unknown ASVs from which five belonged to the Proteobacteria phylum (family Geminicoccaceae); ASV37, ASV70, ASV317, ASV346, ASV507, and ASV1085 (Planctomycetota) (Figure S4, Supporting Information).

#### Fungal communities

Fungal communities presented a total of 1989 ASVs comprised of 10 phyla, 245 genera, and 290 species. The phylum Ascomycota was the most dominant, representing 92% of the fungal relative abundance, followed by Basidiomycota (4%), Glomeromycota (2%), Chrytidiomicota (1%), and Mortierellomycota (1%). Within the Ascomycota, the five most abundant classes were Sordariomycetes (49.5%), Dothiedeomycetes (17.4%), Pezizomycetes (11.6%), Eurotiomycetes (9.5%), and Leoteiomycetes (3.7%) (Fig. 4A). Of the 290 identified species, *Fusarium clamydosporum* accounted for 30% of the fungal relative abundance, followed by three main species; *Preussia terricola* (6%), *Alternaria subcucurbitae* (5.2%), and *Stachybotrys chartarum* (5%). Only 33 species presented a relative abundance higher than 0.5%. PERMANOVA test indicated that only microbial inoculation significantly impacted the communities but not the herbivory stress (Table 3). *Post hoc* pairwise tests between microbial treatments showed that all microbial inoculants significantly impacted the beta diversity in comparison to noninoculated Control plants (Table 3). Also, SynCom’s beta diversity significantly differed from Ba-, Pa-, and Th-inoculated plants, showing a different effect of these three microbes when inoculated as single species. A pairwise comparison of communities of each treatment at different stress conditions showed that only Ba-inoculated plants were significantly altered by the herbivory stress (Ba H− vs. H+) (Table 3).

**Table 3.** Fungal beta diversity. PERMANOVA test values for the effect of microbial inoculation and stress on fungal communities. Pairwise test values for differences between fungal communities.

Fungal communities			
PERMANOVA	F model	*R* ^2^	Pr(> F)
Microbial treatment	2.1809	0.14 179	0.001
Herbivory stress	1.0948	0.0154	0.3
**Pairwise** **^1^**	**F model**	***P*-value**	***P*** **-adjusted**
**Between treatments**			
Control vs. SynCom	4.148	.0001	0.000
Control vs. Ri	3.595	.0001	0.000
Control vs. Th	3.070	.0001	0.000
Control vs. Pa	2.213	.0004	0.001
Control vs. Ba	1.925	.0168	0.026
SynCom vs. Pa	2.185	.0002	0.001
SynCom vs. Ba	3.405	.0001	0.000
SynCom vs. Th	2.507	.0004	0.001
**Within treatment**			
Ba H+ vs. H−	2.637	.0064	0.006

1 Pairwise Adonis values for comparisons between treatments (only statistically significant comparisons reported for Control and SynCom). Pairwise comparison test values within treatment for comparisons of one microbial treatment between two stress conditions (nonstressed; H−, herbivory-stressed; H+). Only significant comparisons are reported.

**Figure 3.**
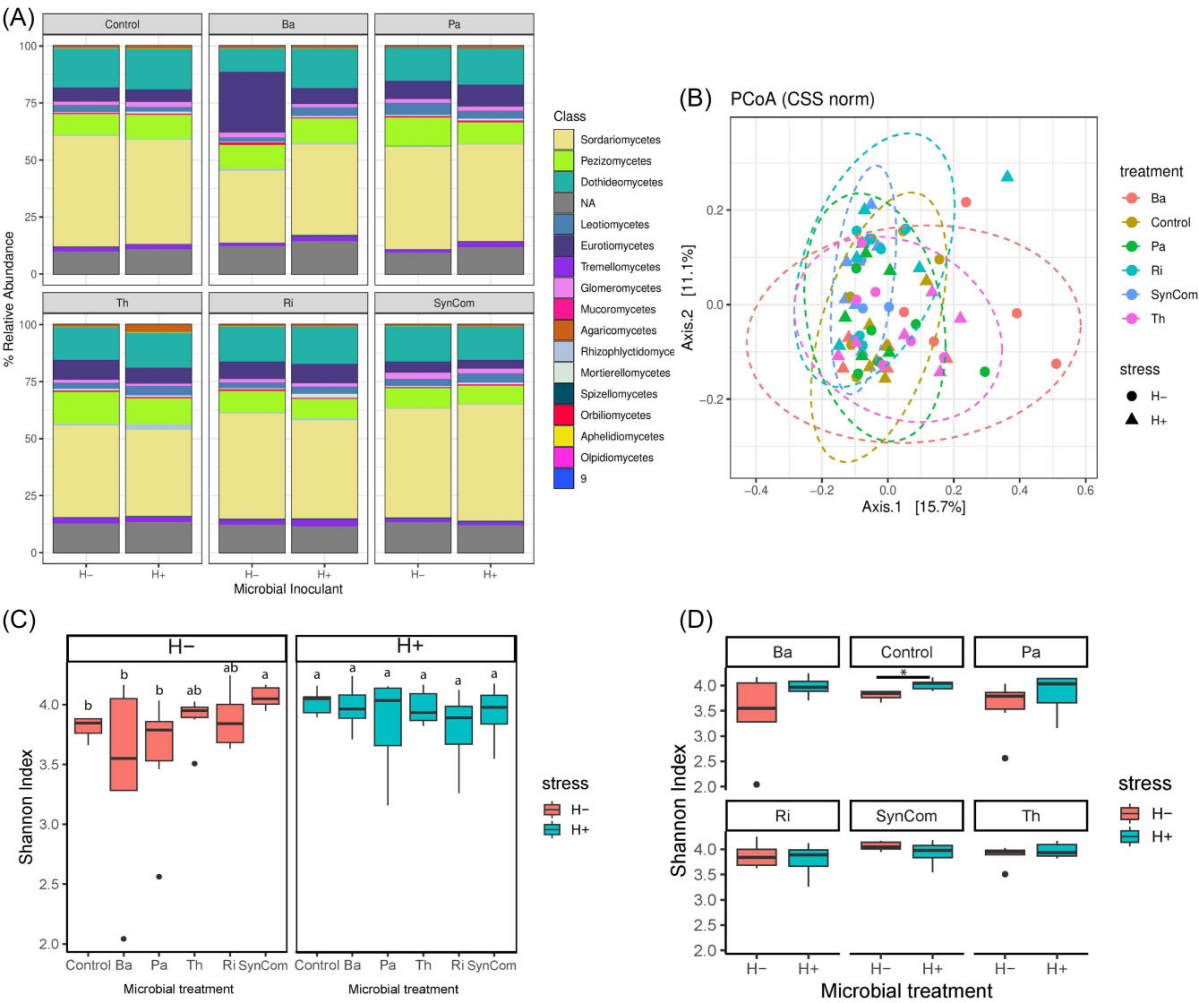
Impact of microbial inoculation and herbivory on fungal communities and diversity. Fungal relative abundance (A), beta diversity (B), and alpha diversity (C) and (D) of tomato rhizosphere communities per microbial treatment; noninoculated (Control), *B. amyloliquefaciens* (Ba), *P. azotoformans* (Pa), *T. harzianum* T22 (Th), *R. irregularis* (Ri), and the SynCom, with (H+) and without herbivory (H−) stress. (A) Bar plot of fungal classes relative abundance grouped by microbial inoculant treatment. Each stacked bar represents the average relative abundance of the different taxa (color guide on the legend) according to the herbivory stress (the left bar for H−; nonstressed, the right bar for H+; herbivory-stressed). (B) PCoA plot of Euclidean distances representing community dissimilarities between treatments (color guide) and stress (circles for nonstressed, and triangles for stressed plants). The *x*- and *y*-axis represents the components explaining the maximum variability between samples. Box plots of alpha diversity Shannon index for comparisons among microbial treatments within one stress condition (C) or within a microbial treatment (D). Statistical differences between comparisons (*P* < .05) were noted with different letters (C) or with an asterisk (D).

Multivariate analysis showed a low separation among treatments despite the differences in microbial communities due to microbial inoculation confirmed by PERMANOVA analysis. However, an overlap between stressed and nonstressed communities was shown for fungal communities (Fig. 3B). Although a similar trend as in bacterial diversity was observed for fungal alpha diversity, neither the overall impact of microbial inoculation nor herbivory stress was statistically significant (Table S6, Supporting Information). However, under no stress conditions, only SynCom-inoculated plants had a significantly higher alpha diversity than the control (Fig. 3C). Although herbivory stress did not have an overall effect on fungal alpha diversity, in noninoculated Control plants a significant increase was observed under herbivory stress (Control H+ vs. H−; *P* = .004) (Fig. 3D). A complete description of bacterial Shannon index values and additional statistical analysis performed on pairwise differences between treatments under different stress levels for Chao1, Observed, and inverse Simpson indexes are provided in Table S7 (Supporting Information).

Similar to alpha diversity results, there was no microbial inoculation or herbivory stress pattern in fungal ASV contrast analysis. All microbial treatments presented a similar number of enriched and reduced ASVs compared to noninoculated control plants both with and without herbivory stress (Table 4). While there were no commonly reduced fungal ASVs, three fungal species were commonly enriched in all treatments with respect to control, regardless of the stress; ASV6 *Fusarium oxysporum* (Sordariomycetes), unknown ASV48 (Sordariomycetes) and unknown ASV23 (Figure S5, Supporting Information). Under no herbivory, two fungal species were commonly enriched in all microbial treatments; ASV163 *Schizothecium fimbriatum* (Sordariomycetes) and ASV172 *Clareoideoglomus walker* (Glomeromycotes). In particular, Ba-inoculated plants showed significant enrichment of two *Aspergillus spp*. (Eurotiomycetes) ASVs; ASV11 and ASV12. Under herbivory stress, In addition to *F. oxysporum*, ASV48, and ASV23, another three ASVs were commonly enriched in all treatments with respect to control (Figure S6, Supporting Information). Two were Sordariomycetes (ASV210 *Neocosmospora rubicola* and ASV7 *Kernia columnaris*) and one unknown ASV7. Only one ASV29 *Plectosphaerella cucumerina* (Sordariomycetes) was commonly reduced in all treatments compared to the control (Figure S6, Supporting Information).

**Table 4.** Fungal ASVs differential relative abundance. Total numbers of significantly reduced or enriched (fold-changed) ASVs under nonherbivory (H−) or leaf-herbivory stress (H+) in control plants with respect to the compared microbial treatment.

	Nonherbivory (H−)			Herbivory (H+)		
Comparison	Reduced	Enriched	Total	Reduced	Enriched	Total
Control vs. Ba	8	8	16	8	5	13
Control vs. Th	8	5	13	8	6	14
Control vs. SynCom	7	4	11	8	4	12
Control vs. Ri	7	6	13	12	6	18
Control vs. Pa	6	5	11	7	8	15

#### Impact of microbial inoculation on the rhizosphere volatilome

Volatiles from roots inoculated with the six microbial inoculant treatments were compared before (T0) and 24 h after (T1) leaf herbivory stress by *S. exigua*. A total of 24 volatile compounds were detected across treatments (Table 5). The volatiles belonged to nine chemical classes: alcohol, aldehyde, alkane, aromatic, carboxylic acid, furan, monoterpene, a sulfur compound, and thiazole. One-way ANOVA of each volatile compound showed that microbial inoculation had a significant impact on six compounds; acetic acid, benzene acetaldehyde, decanal, dimethyl disulfide, and two unknown compounds (954 and 985) (Table 5). The average peak intensity of unknown compound 954 was significantly higher in bacterial-inoculated treatments (Ba and Pa) and noninoculated control, whereas decanal and benzene acetaldehyde were higher in fungal-inoculated treatments (Ri, Th, and SynCom).

**Table 5.** Tomato rhizosphere volatilome under microbial inoculation and herbivory stress. List of all volatile compounds detected in tomato rhizospheres from both control and inoculated plants with and without herbivory stress. Volatile compounds are classified according to chemical class and sorted by calculated retention index according to HP-5 column type (RI cal) and contrasted to literature (RI lit). Statistical analysis output provided for differences between microbial treatments under nonherbivory conditions (control; C, *B. amyloliquefaciens*; Ba, *P. azotoformans*; Pa, *T. harzianum*; Th, *R. irregularis*; Ri, and synthetic community; SynCom).

Compound	Class	RI cal	RI lit^a^	*P*-value	C	Ba	Pa	Th	Ri	SynCom
Acetic acid	Carboxylic acid	618	610	.007	a	b	ab	a	a	ab
Dimethyl disulfide	Sulfur	747	746	.007	ab	a	b	a	a	c
Octane	Alkane	800	800							
Hexanal	Aldehyde	802	801							
Nonane	Alkane	899	900							
Unknown 954	NA	954	NA	.002	a	a	a	ab	b	ab
Benzaldehyde	Aldehyde	966	692							
DMTS	Sulfur	973	971							
Unknown 985	NA	985	NA	.004	a	a	a	a	b	a
Benzonitrile	Aromatic	988	984							
2-pentyl furan	Furan	989	993							
Benzofuran	Furan	999	1000							
Octanal	Aldehyde	1003	1003							
p-Cymene	Monoterpene	1027	1025							
2-ethyl-1-hexanol	Alcohol	1028	1030							
Limonene	Monoterpene	1031	1031							
Benzyl alcohol	Alcohol	1034	1036							
β-Phellandrene	Monoterpene	1034	1031							
Benzene acetaldehyde	Aldehyde	1047	1045	.006	a	a	a	b	b	b
Acetophenone	Ketone	1069	1066							
Nonanal	Aldehyde	1105	1104							
Unknown1190	NA	1190	NA							
Decanal	Aldehyde	1207	1206	.005	a	a	a	ab	b	ab
Benzothiazole	Thiazole	1235	1228							

a RI lit. Reference retention index value used for identification according to NIST 2020 database.

**Figure 4.**
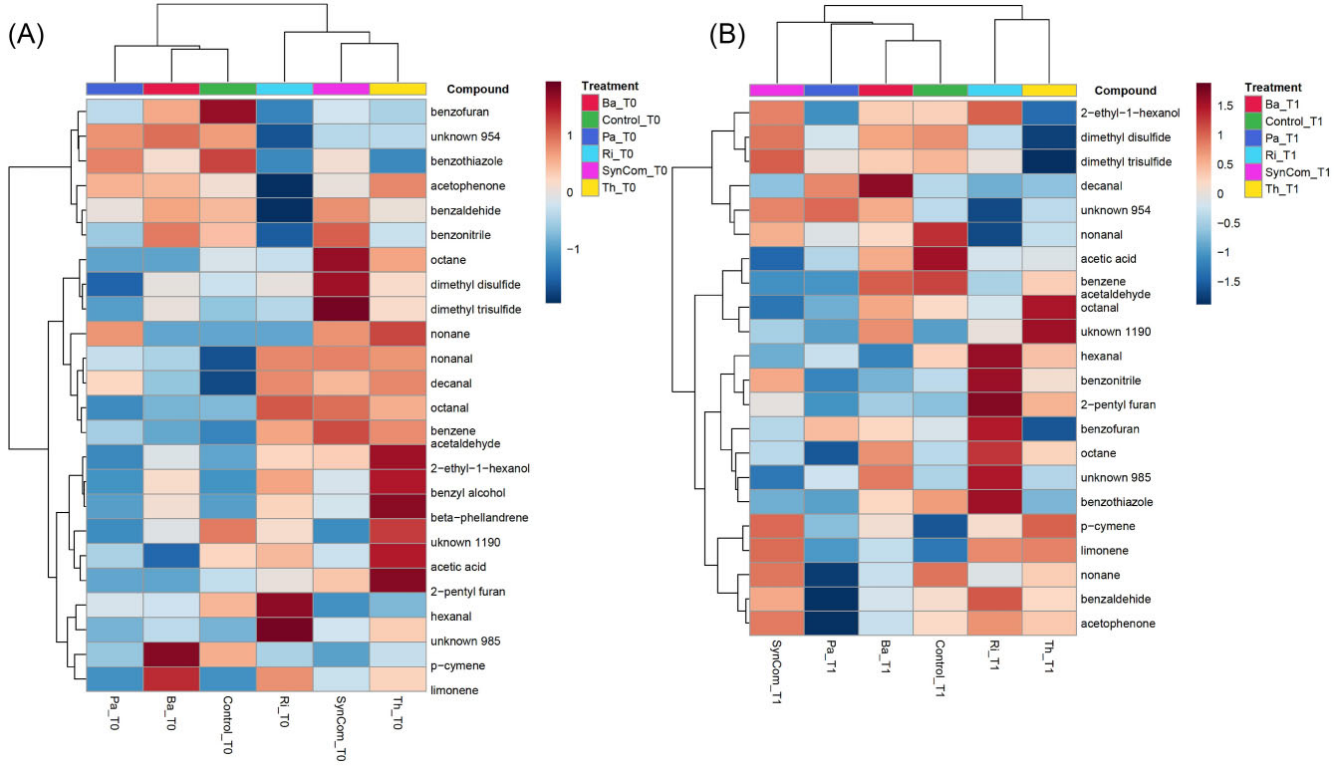
Impact of microbial inoculation on rhizosphere volatilome with and without herbivory stress. Heatmaps of volatile compounds’ peak intensity averaged (*n* = 6) per microbial treatment (Control, Ba, Pa, Th, Ri, and SynCom). Left panel (A) compares rhizosphere volatilomes among microbial treatments without stress (T0). Right panel (B) compares rhizosphere volatilomes among microbial treatments upon 24 h of leaf herbivory stress (T1). Compound peak intensity is indicated with a color gradient legend with dark blue as the lowest intensity and dark red as the highest intensity.

Hierarchical clustering according to Euclidean distances of treatment averages showed that fungal inoculated treatments (Ri, Th, and SynCom) grouped, while the bacterial treatments were more similar to noninoculated Control plants (Fig. 4). Interestingly, after the stress induction, one-way ANOVA analysis showed that no volatile compounds were found to be significantly different among treatments. Therefore, the hierarchical clustering of treatment averages did not group according to the microbial treatment, therefore the SynCom did not cluster together with the rest of the fungal treatments (Ri and Th) (Fig. 4).

**Figure 5.**
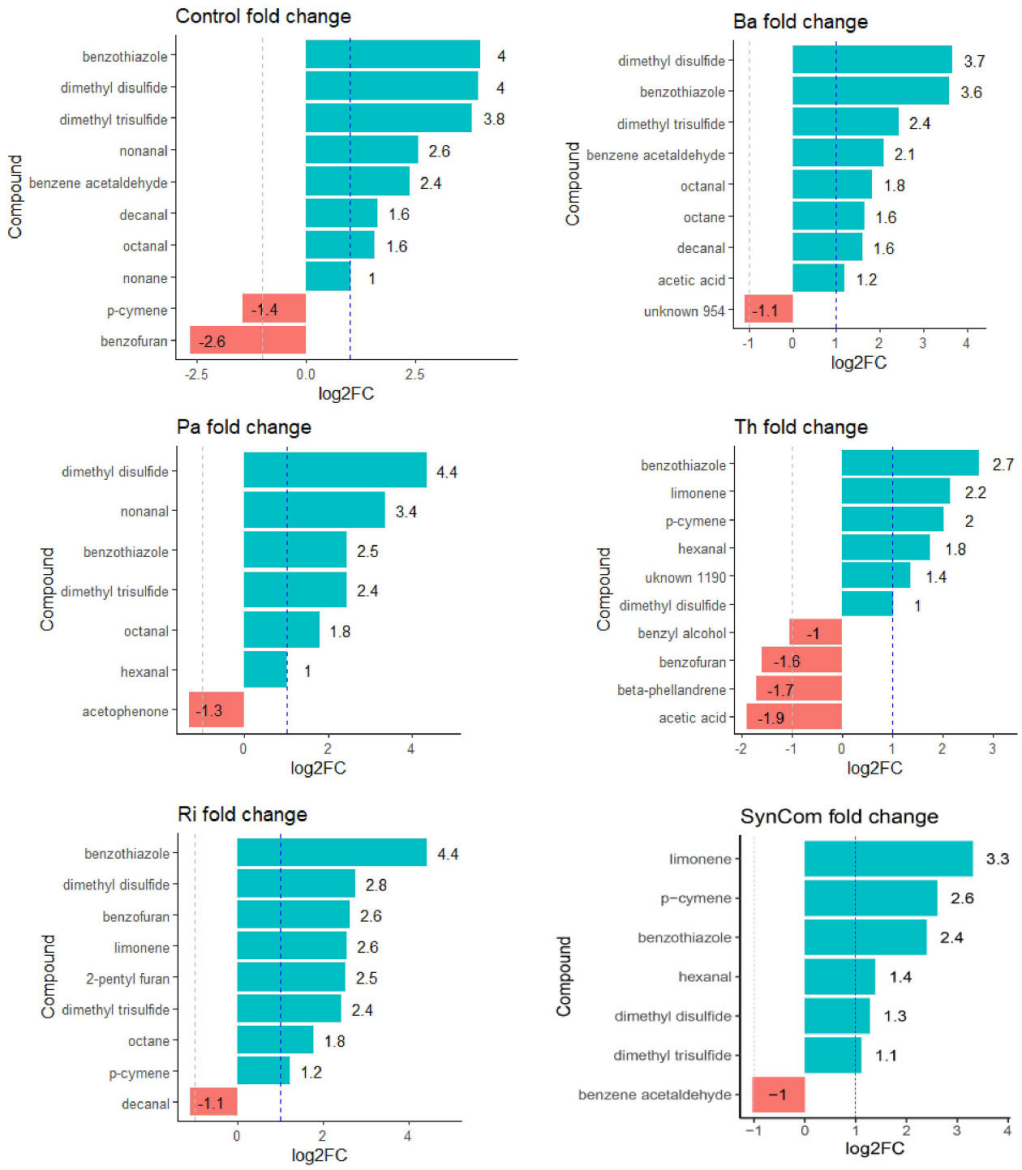
Effect of leaf herbivory stress on rhizosphere volatilomes. Bar plots of significantly changed volatile compounds’ peak intensities after 24 h of shoot herbivory stress within each microbial treatment. The dashed line indicates a 2-fold logarithmic change (log2 FC) peak intensity increase under herbivory stress with respect to the previous nonstressed condition. FC values next to horizontal colored bars for each compound; blue for increased compounds under herbivory and red for reduced compounds under herbivory).

FC analysis of the value change between subsequent measurements showed significant changes in all treatments before and after herbivory stress, with an increase of two volatile compounds in all treatments: benzothiazole and dimethyl disulfide (DMDS). The majority of significantly different compounds increased under herbivory stress with respect to the control condition (Fig. 5). The classes of stress-enhanced compounds were mainly sulfur, alkane, aldehyde, benzene, furan, and monoterpene. Dimethyl trisulfide (DMTS) significantly increased under stress in all treatments except for Th. A pattern was shown between bacterial and fungal inoculants: bacterial inoculants (Ba and Pa) together with control plants showed an increase of alkanes and aldehydes; either from hexanal, octanal, octane, nonanal, nonane, or decanal. The fungal inoculants (Ri, Th, and SynCom) shared a FC increase of monoterpenes such as p-cymene and limonene. Particularly, Ri-inoculated plants were the only ones presenting a fold-increase of the two furan compounds benzofuran and 2-pentyl furan. Decreased FC compounds were different across all treatments. The Th treatment showed the largest number (four) of reduced compounds under herbivory (Fig. 5).

## Discussion

Our study aimed to understand the impact of bacterial and fungal inoculants on the rhizosphere microbiota composition and volatilome of tomato plants under herbivory stress. First, we hypothesized that microbial inoculants would impact both the rhizosphere microbial community composition and the rhizosphere volatilome. We revealed that the individual bacterial and fungal inoculants and the SynCom had distinctive impacts on the rhizosphere microbiome composition differing among themselves and in comparison, to the noninoculated control plants. Also, rhizosphere volatiles were impacted upon inoculant treatment, showing each microbial inoculant treatment a unique blend of volatile compounds among themselves and compared to control plants. Next, we hypothesized that the addition of a continuous leaf herbivory stress to a plant–microbial inoculant system would impact the relationship of the plant and the microbial inoculants both at rhizosphere microbiome and volatilome level. We confirmed that herbivory stress had a general effect irrespective of the inoculant type for the majority of measured parameters on rhizosphere microbiome composition, although some effects were stress-inoculant specific. Also, under herbivory each microbial inoculant presented different volatile blends compared to their previous nonstressed condition. Our study confirmed that the effects of the five different microbial inoculants and the microbial consortium on plant physiology can be general or stress-dependent. There was not major impact on plant physiology traits such as shoot nutrient content or biomass due to the inoculation. However, we observed that herbivory had a general significant effect on leaf nutrient content and a microbe-dependent increase of N in noninoculated control and Ba-inoculated plants. These two treatments showed a significantly higher relative amount of shoot N (therefore a significantly different C/N ratio) under herbivory stress. The increase in N in the shoot might be related to the mobilization of amino acids for the synthesis of defense-related compounds (Zhou et al. 2015). Complementary analysis on the pest relative growth or feeding performance would be necessary to determine the positive or negative impact on the pest of the N increase. Regarding the shoot biomass, neither the inoculants nor the herbivory had a significant effect, except for Ri-inoculated plants, where a significant reduction of shoot biomass was observed under herbivory. Similarly, further studies on the pest performance might help clarify whether the biomass reduction is caused due to higher consumption rate by the pest or a growth-defence trade-off (Züst and Agrawal 2017). Overall, these results indicate that the impact of microbial inoculants and the herbivory stress was unique for each plant–microbial inoculant–stress combination, indicating a certain level of specificity in each tripartite plant–microbe–insect interaction.

Amplicon sequencing analysis of rhizosphere soils showed that all microbial inoculants and the herbivory stress impacted differently the bacterial and fungal communities in terms of diversity and structure. Bacterial alpha diversity was impacted by the microbial inoculants only under no-stress conditions. The diversity of *B. amyloliquefaciens* (Ba)- and *T. harzianum* T22 (Th)-inoculated plants were the lowest compared to control. It is unclear whether these inoculants directly competed with the local microbiota altering the rhizosphere through microbe–microbe interactions, or indirectly altered the plant’s physiology through ISR activation. It has been shown that the coinoculation of *Bacillus* spp.- and *T. harzianum*-induced changes in the rhizosphere creating a suppressive environment against a pathogenic *Fusarium* strain (Xiong et al. 2017). On the other hand, inoculated microbes can alter the plant’s phytohormone balance and affect the plant’s responses to later colonizers (Toju et al. 2018). However, such effects on the alpha diversity were no longer observed upon leaf herbivory stress. Aboveground stresses can cause dramatic changes in plant physiology leading to differential microbial recruitment via changes in root exudates and volatiles (Yi et al. 2011). Although the alpha diversity was overall increased under stress, all treatments showed similar levels of diversity, indicating that the herbivory nullifies or evens the effect of the individual inoculants. Although bacterial communities were dominated by Actinobacteria and Proteobacteria, among other phyla typically reported as rhizosphere-associated bacteria (Trivedi et al. 2020), differences in relative ASVs abundance between treatments compared to control were more pronounced under stress. Inoculants Ba and Th presented the largest number of differentially abundant ASVs with respect to noninoculated control plants (both with and without herbivory). Under nonstress conditions, they showed a common reduction with respect to noninoculated control plants of the actinobacterium *Cellulomonas* spp., which has been previously reported to be highly associated with tomato landraces typical from southern Spain (Smulders et al. 2021). Together with the SynCom, these three treatments presented a common enrichment of diverse bacterial genera; *Blastococcus* spp. (Actinomycota) has been reported in the rhizosphere of tomato plants adapted to arid, desert, or stone environments (Sghaier et al. 2016), and *Ramilibacter* spp. (Pseudomonadota) and *Ohtaekwangia* spp. (Bacteroidetes) were reported in grapevine fields related to unfarmed soils or recently farmed soils (Liu et al. 2021). However, the role these microbes have in association with tomato plants is still unknown. Under herbivory stress, a general herbivory effect was observed since a higher number of ASVs were commonly enriched among microbial treatments compared to noninoculated control plants. This indicated that the microbial recruitment upon stress was not impaired or reduced by the microbial treatments, but had a different recruitment outcome than in noninoculated control plants. However, most of the commonly recruited microbes by the treatments under stress were unknown ASVs. The beta diversity of bacterial communities was more affected by the microbial treatments. The ASVs recruited under stress may be part of the rare rhizosphere microbiome, which is known to have an important role in microbial communities despite its low relative abundance (Jia et al. 2018).

Fungal alpha diversity was generally not affected by microbial inoculation or herbivory. Although a trend was visible like in bacterial alpha diversity, only under control conditions there are differences between treatments that are no longer detectable under stress. Under no stress, SynCom was the treatment with the highest alpha diversity. Similarly, SynCom showed the most different fungal communities with respect to control. Beta diversity of fungal communities was largely affected by the microbial inoculation regardless of the stress. In general, fungal communities were dominated by Ascomycota (more than 90% relative abundance), in particular by the Sordariomycetes order, and the single species *Fusarium chlamydosporum* accounted for one-third of the relative abundance. Like many other *Fusarium* spp. endophytic strains that have been reported to be beneficial to plants (Pappas et al. 2018, de Lamo and Takken 2020, Toghueo 2020), *F. chlamydosporum* can be a nonpathogenic free-living strain with necrotrophic behavior (Torbati et al. 2021). Interestingly the Ba-inoculated plants presented a larger abundance of *Aspergillus* spp. under no-herbivory conditions than under herbivory stress. Plants can shift from one microbial partner to another under stress (Zuccaro 2020) and it is possible that under herbivory stress some plant–microbe relationships might be altered. Although the *Aspergillus* species was unknown, it is possible that it was recruited for P-solubilization capacity (Khan et al. 2007) since the plants were watered with a P-limited fertilization regime. This general microbial-treatment effect was also observed in similar patterns in terms of ASVs relative abundance. Microbial inoculation effects on fungal communities were general since all treatments had enrichment of *F. oxysporum* compared to control plants. It is possible that the microbial inoculation and stress had a systemic effect on the plant defenses and phytohormonal balance (Friman et al. 2021) that created a suitable niche for *F. oxysporum* (Constantin et al. 2019). In addition to *F. oxysporum*, other fungal species were commonly enriched in all treatments upon herbivory stress with respect to control. Three ASVs reported as unknown taxa, *Kernia* spp., which has been reported as a decomposer of herbivore dung (Caretta et al. 1998), and *Neocosmospora rubicola*, reported as both plant pathogen (Zheng et al. 2018, Riaz et al. 2022) and as plant endophyte together with other species from the genus (Kim et al. 2017, Sandoval-Denis et al. 2019). However, whether these fungal species benefit from the plant’s stress condition or play an active role in helping the plant’s defenses is unknown. In general, fungal effects are mainly dominated by microbial inoculation and are general, meaning that microbial inoculants have less pronounced individual effects. The arid nature of the region where the soil was extracted could explain the high abundance of *Fusarium* species, known to thrive in desertic and extreme environments (Mandeel 2006).

Inoculated plants with the SynCom behaved differently than the other microbial treatments and the noninoculated controls. Individually inoculated Ba or Th reduced bacterial alpha diversity while SynCom-inoculated plants did not. Also, SynCom bacterial beta diversity was different from noninoculated control and to Ba- and Th-inoculated plants. For fungal communities, SynCom inoculated plants had higher alpha diversity and the beta diversity differed from control and Ba-, Pa-, and Th-inoculated plants. However, it is not clear what are the microbial interactions between the inoculated microbes or whether some microbes have a more predominant role than others. Plant shoot nutrient and biomass data indicated that SynCom did not have an additional effect. It is questioned whether microbial consortia translate into additional beneficial effects for the plants (Bradáčová et al. 2019b). Microbial consortia are often reported to deliver better effects under stress (Bradáčová et al. 2019a, Joshi et al. 2020).

Microbial inoculants impacted the rhizosphere volatilome under the nonstress condition. Six volatiles were found to be different among treatments only by inoculation. All six compounds were significantly higher in one or more fungal-inoculated treatments (Th, Ri, and SynCom). Interestingly, the SynCom behaved more similarly to the fungal inoculants Ri and Th in terms of rhizosphere volatilome. Th-inoculated plants showed a high intensity of acetic acid, which might be related to the indole-3-acetic acid production reported in many *Trichoderma* strains as a root growth-promoting hormone (Nieto-Jacobo et al. 2017). Ri-inoculated plants showed an increase in unknown 985 and SynCom-inoculated plants in DMDS. Upon stress, an overall volatilome change was observed with no volatile compound significantly different between treatments. Two compounds were commonly increased in all treatments upon stress: DMDS and benzothiazole. Both DMDS and benzothiazole have been reported as microbial volatiles with antifungal or antimicrobial properties. For example, DMDS has been shown to effectively reduce *F. oxysporum* populations (Papazlatani et al. 2016) while benzothiazole has antimicrobial functions against pathogens (Gao et al. 2017, Lammers et al. 2021). It has been shown that aboveground herbivory can reduce the root volatile production of terpenes and increase the production of aldehydes and sulfur compounds (Lee-Diaz et al. 2022). Bacterial inoculants showed similar patterns in volatile enrichment upon stress: by increasing the production of sulfur compounds, aldehydes, and alkanes. However, fungal inoculants and the SynCom behaved similarly by increasing the production of terpenes, alcohols, and furans. Despite the similarities, each microbial inoculant had a unique “stress-enriched” volatilome; Ri-inoculated plants showed enrichment in two furan compounds, which are mainly produced by fungi and have been related to antimicrobial functions (Farh and Jeon 2020), while Th-inoculated plants presented both increase and decrease of monoterpenes and furan compounds. The unique stressed rhizosphere volatilome of each microbial treatment could have a further impact on ecological interactions with other soil trophic levels. For example, increased DMDS from stressed roots has been shown to attract natural enemies of root-feeding herbivores (Danner et al. 2015). Also, aldehydes have been reported as attractants to soil-dwelling insects, such as coleopteran larvae (Barsics et al. 2017).

Overall, our results showed that aboveground herbivory affected the rhizosphere’s volatile and microbiome composition. This stress-associated effect was impacted by microbial inoculation, and depending on the parameter measured (diversity, community structure, biomass, and colonization), the effects were common for all microbial inoculants or inoculant-specific. Overall, the impact of microbial inoculation was stronger under nonstress conditions. The differences found in both community structure and volatilome were leveled by herbivory, illustrating that biotic stress is a major driver of plant–microbe interactions. However, the unique interaction of microbial inoculant-herbivory-dependent effects reflects the complexity of the plant’s ecological interactions and context dependency. Further knowledge on the ecological role of inoculants and their interactions with biotic and abiotic factors can bring knowledge that improves their efficacy and safe application.

## Supplementary Material

fiad160_Supplemental_File
