# Supplementary material for: Impact of bacterial and fungal inoculants on the resident rhizosphere microbiome and the volatilome of tomato plants under leaf herbivory stress
Source: FEMS Microbiol Ecol. 2024 Feb 8;100(2):fiad160. doi: 10.1093/femsec/fiad160 (PMC10858387; doi:10.1093/femsec/fiad160)
Supplement: fiad160_Supplemental_File [file fiad160_supplemental_file.docx]

**Impact of bacterial and fungal inoculants on the resident rhizosphere microbiome and the volatilome of tomato plants under leaf herbivory stress**

**Supplementary Information**

|  |  | Page |
| --- | --- | --- |
| **Table S1.** | Difco Sporulation Medium (DSM) | 1 |
| **Table S2.** | Spodoptera exigua artificial diet recipe. | 2 |
| **Table S3.** | MzMine v.2.53 parameters for peak intensity tables. | 3 |
| **Figure S1.** | Mycorrhization percentage of tomato roots after 8 weeks. | 4 |
| **Figure S2** | Bar plots of shoot dry biomass. | 5 |
| **Table S4.** | Analysis of C, N and C/N ratio shoot nutrient content. | 6 |
| **Table S5.** | Alpha diversity values and statistical analysis of bacterial rhizosphere communities. | 7 |
| **Figure S3** | ASVs differential analysis of bacterial communities vs Control under no herbivory (H-). | 8 |
| **Figure S4.** | ASVs differential analysis of bacterial communities vs Control under herbivory (H+). | 9 |
| **Table S6.** | Effect of microbial inoculation treatment and herbivory stress in alpha diversity Shannon index of fungal rhizosphere communities according to Kruskal-Wallis test. | 10 |
| **Table S7.** | Alpha diversity values and statistical analysis of fungal rhizosphere communities. | 11 |
| **Figure S5.** | ASVs differential analysis of fungal communities under no herbivory (H-). | 12 |
| **Figure S6.** | ASVs differential analysis of fungal communities under herbivory (H+). | 13 |
| **Table S8.** | Rhizosphere volatile compounds of tomato plants before leaf herbivory (T0) and 24 h after leaf herbivory stress (T1). | 14 |

**Table S1.** Difco Sporulation Medium (DSM)

| Difco Sporulation Medium (DSM) | | | | |
| --- | --- | --- | --- | --- |
| Composition: | |  |  |  |
| Solution | **Compound** | **Quantity** | **Unit** | **Per** |
| A | Nutrient broth (Oxoid) | 8 | g | 1 L |
|  | KCl | 1 | g | 1 L |
| B | MgSO4.7H2O | 1,2 | g | 100 mL |
| C | Ca(NO3)2.4H2O | 23,61 | g | 100 mL |
| D | MnCl2.2H2O | 161,9 | mg | 100 mL |
| E | FeSO4.7H2O | 27,8 | mg | 100 mL |
| Preparation: |  |  |  |  |
| 1. Dissolve medium components of solution A in 900 mL. | | | |  |
| 3. Add 10 mL of solution B to A. | |  |  |  |
| 4. Adjust solution A to 1 L, adjust pH to 7.6 and Autoclave. | | | |  |
| 5. Add 1 mL of sterile solutions C, D, and E to solution A. | | | | |

**Table S2.** *Spodoptera exigua* artificial diet recipe.

| Ingredients | Amount |
| --- | --- |
| Tap water | 1 liter |
| Agar | 18 g |
| Corn flour | 96 g |
| Wheat germ | 64 g |
| Yeast | 34 g |
| Casein | 10 g |
| Wesson salts mixture | 5 g |
| Ascorbic acid | 9 g |
| Vitamin mix^1^ | 0.06 g |
| Sorbic acid (99%) | 2.5 g |
| Tetracycline | 0.5 g |
| Nipagin | 1 g |
| Corn oil | 4 ml |

^1^Vitamin mix: nicotinic acid (6 g), riboflavin (3 g), thiamin (1.5 g), pyridoxine (1.5 g), Ca-pantothenate (12 g), folic acid (3 g), biotin (0.12 g), cyanocobalamin (0.012 g).

Preparation

Mix 1 liter of water with the corn oil and bring to a boil. Cool down until 55-60°C and add the remaining ingredients. Stir vigorously with a mixer until to reach a dough-like consistency. Store in individual containers and freeze at -20°C until usage.

**Table S3.** MzMine v.2.53 parameters for peak intensity tables.

| **Step** | **Parameter [unit]** | **Value** |
| --- | --- | --- |
| Mass Detection (centroid) | Retention time range  Noise Level | 1-16min  1.00E+03 |
| Chromatogram Building (ADAP) | Minimum group size [unit]  Group intensity threshold  m/z Tolerance [mz / ppm] | 5  1.00E+03  0.01/ 5 |
| Peak Deconvolution (Wavelets ADAP) | S/N threshold  Minimum feature height  Coefficient/area threshold  Peak duration range [min]  RT wavelet range [min] | 10  1.00E+03  200  0-1.5  0-0.1 |
| Spectral Deconvolution  (Hierarchical clustering ADAP) | Minimum cluster distance  Minimum cluster size  Minimum cluster intensity  Minimum edge-to-height ratio  Minimum delta-to-height ratio  Minimum sharpness  Shape similarity tolerance | 0.01  1  1.00E+04  0.3  0.2  10  18 |
| Alignment (ADAP aligner GC) | Minimum confidence  Retention time tolerance (min)  m/z Tolerance [mz/ppm]  Score threshold  Score weight | 0.7  0.1  0.01/5  0.75  0.1 |
|  |  |  |
|  |  |  |

**Figure S1.** Mycorrhization percentage of tomato roots after 8 weeks. Microbial inoculant treatments compared are Control (no microbial inoculation), Ri (*R. irregularis*), and SynCom (contains *R. irregularis*). Left panel in red shows plants without herbivory stress (H-), while the right panel in blue shows plants stressed with herbivory for two weeks (H+).

**
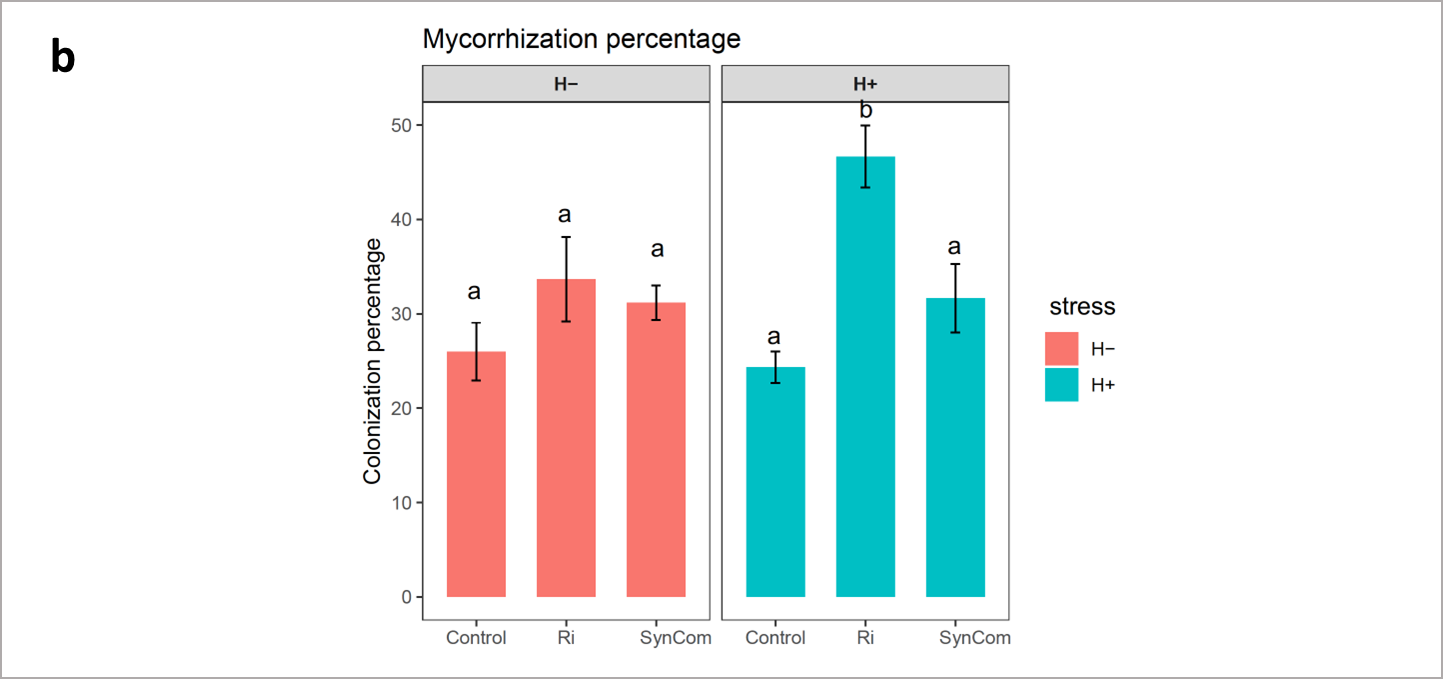
**

**Figure S2.** Bar plots of shoot dry biomass. Shoot biomass (mg) of inoculated tomato plants (non-inoculated Control, Ba, Pa, Th, Ri and SynCom) under two leaf herbivory stress levels; H+; stressed and H-; non-stressed.


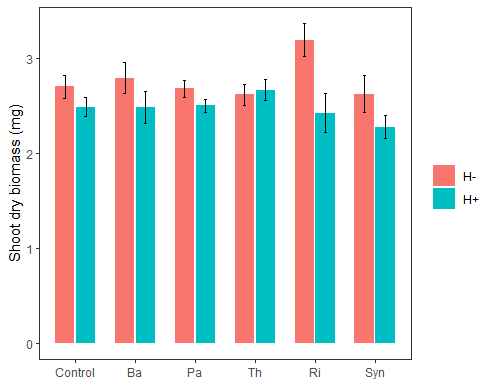


**Table S4.** Analysis of C, N, and C/N ratio shoot nutrient content. Statistical analysis of the impact of microbial inoculation (Treatment) and stress (Herbivory) on the relative content of Carbon (C), Nitrogen (N), and C/N ratio in tomato shoot and mean values (in µg/mg of sample tissue) with standard deviation (sd) for each variable. Analyses output for; (1) general effects (2-way ANOVA), (2) effects of microbial treatments within one herbivory condition (between treatments), and (3) effects of herbivory within each microbial treatment (within treatments). Statistical differences are indicated with an asterisk (p<0.05) and Post-hoc analysis is indicated with different letters.

|  |  | |  | |  | |  |  |  |
| --- | --- | --- | --- | --- | --- | --- | --- | --- | --- |
|  | **CARBON** | | | **NITROGEN** | | | | **CN** | |
| **2-way ANOVA** | **F-value** | **Pr (>F)** | | **F-value** | | **Pr (>F)** | | **F-value** | **Pr (>F)** |
| Treatment | 2.192 | 0.067 | | 0.136 | | 0.983 | | 0.297 | 0.913 |
| Herbivory | 5.441 | 0.023* | | 2.971 | | 0.090 | | 6.157 | 0.016* |
| Treatment: Herbivory | 2.860 | 0.022* | | 4.231 | | 0.002** | | 3.506 | 0.008** |
| *Between treatments* | **Mean ± sd** | **Sig** | | **Mean ± sd** | | **Sig** | | **Mean ± sd** | **Sig** |
| *Non-herbivory* |  |  | |  | |  | |  |  |
| Control | 38.6 ± 0.29 | ab | | 0.82 ± 0.07 | | bc | | 47.34 ± 4.11 | a |
| Ba | 38.61 ± 0.26 | ab | | 0.81 ± 0.03 | | c | | 47.89 ± 1.8 | a |
| Pa | 37.87 ± 1.23 | b | | 0.84 ± 0.03 | | abc | | 44.94 ± 1.88 | a |
| Th | 39.91 ± 2.03 | a | | 0.92 ± 0.12 | | a | | 43.66 ± 4.29 | a |
| Ri | 38.53 ± 0.3 | ab | | 0.87 ± 0.03 | | a | | 44.19 ± 1.46 | a |
| SynCom | 38.27 ± 0.84 | ab | | 0.87 ± 0.06 | | ab | | 44.16 ± 3.07 | a |
| *Herbivory* |  |  | |  | |  | |  |  |
| Control | 38.05 ± 0.51 | ab | | 0.93 ± 0.07 | | a | | 41.23 ± 3.26 | a |
| Ba | 38.45 ± 0.18 | ab | | 0.92 ± 0.06 | | a | | 41.78 ± 2.42 | a |
| Pa | 38.51 ± 0.46 | a | | 0.87 ± 0.06 | | a | | 44.39 ± 3.53 | a |
| Th | 38.19 ± 0.66 | ab | | 0.83 ± 0.04 | | a | | 46.3 ± 2.62 | a |
| Ri | 37.49 ± 0.73 | b | | 0.87 ± 0.06 | | a | | 43.06 ± 2.91 | a |
| SynCom | 38.25 ± 0.56 | ab | | 0.87 ± 0.08 | | a | | 44.1 ± 4.56 | a |
| *Within treatments* |  | **p.val** | |  | | **p.val** | |  | **p.val** |
| Control H- vs H+ |  | 0.052 | |  | | 0.029* | |  | 0.018* |
| Ba H- vs H+ |  | 0.284 | |  | | 0.006** | |  | 0.002** |
| Pa H- vs H+ |  | 0.394 | |  | | 0.324 | |  | 0.144 |
| Th H- vs H+ |  | 0.093 | |  | | 0.179 | |  | 0.232 |
| Ri H- vs H+ |  | 0.065 | |  | | 0.969 | |  | 0.419 |
| SynCom H- vs H+ |  | 0.818 | |  | | 0.916 | |  | 0.979 |

**Table S5.** Alpha diversity values and statistical analysis of bacterial rhizosphere communities. Mean and standard deviation values of inoculated plants under herbivory and no-herbivory stress. Alpha diversity indexes Chao 1, Inverse Simpson and Observed were tested for differences between treatments with one-way ANOVA. Within each stress condition, post-hoc Tukey HSD tests confirmed significant differences (p<0.05) between treatments (groups) are indicated with different letters.

|  | **Chao1** |  | **Inv. Simpson** | |  | | | **Observed** | |  | |  |  |
| --- | --- | --- | --- | --- | --- | --- | --- | --- | --- | --- | --- | --- | --- |
| **Treatment** | **Mean ± sd** | **grp** | | **Mean ± sd** | | **grp** | **Mean ± sd** | | **grp** | |  |  |  |
| *No herbivory* | | | | | | | | | | | | |  |
| Control | 716.65 ± 61.21 | a | | 283.34 ± 37.79 | | a | 690.67 ± 56.37 | | a | |  |  |  |
| Ba | 566.45 ± 102.69 | ab | | 219.09 ± 33.78 | | a | 551 ± 93.29 | | ab | |  |  |  |
| Pa | 731.47 ± 131.46 | a | | 296.29 ± 63.14 | | a | 702.71 ± 114.15 | | a | |  |  |  |
| Th | 505.32 ± 131.1 | b | | 222.24 ± 43.81 | | a | 495.17 ± 123.43 | | b | |  |  |  |
| Ri | 696.32 ± 116.43 | a | | 275.03 ± 56.23 | | a | 666.67 ± 100.69 | | a | |  |  |  |
| SynCom | 638.01 ± 75.25 | ab | | 270.57 ± 37.44 | | a | 616.83 ± 67.97 | | ab | |  |  |  |
| *Herbivory* | | | | | | | | | | | | | |
| Control | 765.78 ± 66.31 | a | | 293.55 ± 30.46 | | a | 734.67 ± 58.51 | | a | |  |  |  |
| Ba | 668.62 ± 88.84 | a | | 275.12 ± 29.65 | | a | 645.17 ± 80.78 | | a | |  |  |  |
| Pa | 699.8 ± 111.4 | a | | 258.91 ± 90.14 | | a | 673.67 ± 100.7 | | a | |  |  |  |
| Th | 638.1 ± 44.31 | a | | 246.14 ± 36.48 | | a | 615.33 ± 37.85 | | a | |  |  |  |
| Ri | 760.1 ± 85.28 | a | | 296.31 ± 58.31 | | a | 726.67 ± 77.8 | | a | |  |  |  |
| SynCom | 744.18 ± 83.64 | a | | 281.6 ± 48.17 | | a | 707.17 ± 75.9 | | a | |  |  |  |


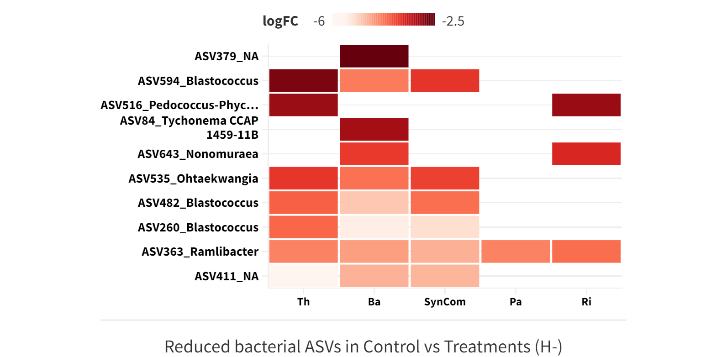

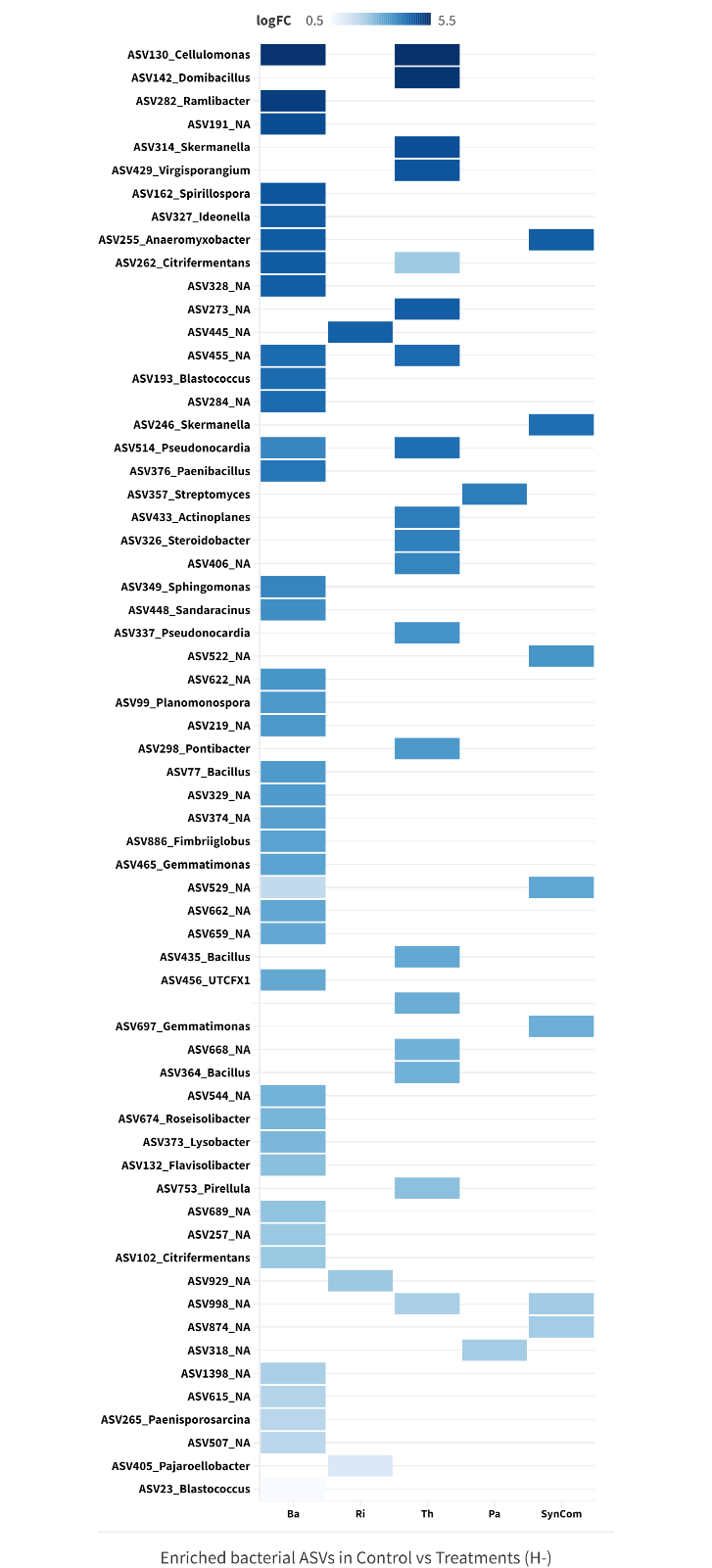
**Figure S3.** ASVs differential analysis of bacterial communities vs Control under non-herbivory (H-); (a) enriched in Control (blue) and (b) reduced in Control (red).

**
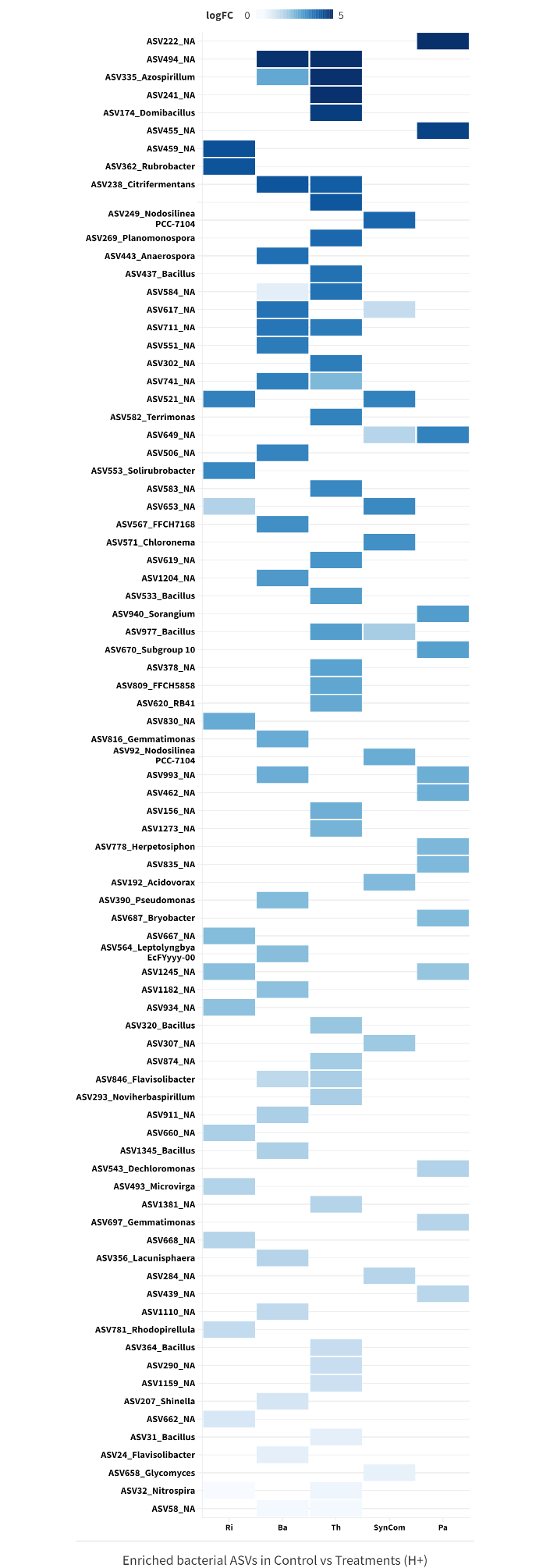

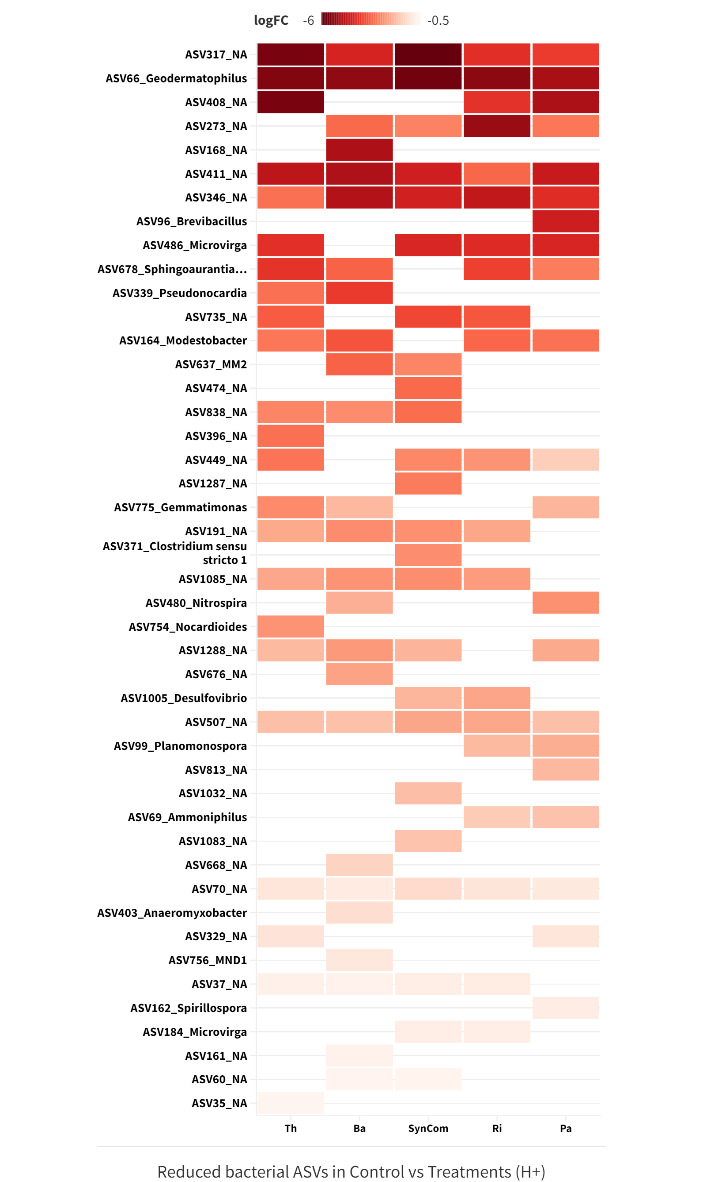
Figure S4.** ASVs differential analysis of bacterial communities vs Control under herbivory (H+); (a) enriched in Control (blue) and (b) reduced in Control (red).

**Table S6.** Effect of microbial inoculation treatment and herbivory stress in alpha diversity Shannon index of fungal rhizosphere communities according to Kruskal-Wallis test. Statistical output (Chi square, p-chisq and significance codes) provided for factors tested individually and in interaction.

| *Shannon index fungal communities* | | | |
| --- | --- | --- | --- |
| **Kruskal-Wallis** | **Chisq** | **p.chisq** | **Sig^1^** |
| Microbial treatment | 5.035 | 0.4116 |  |
| Herbivory stress | 2.781 | 0.0954 |  |

^1^ Significance codes: 0 ‘***’ 0.001 ‘**’ 0.01 ‘*’ 0.05 ‘.’ 0.1 ‘ ’ 1 *-*

**Table S7.** Alpha diversity values and statistical analysis of fungal rhizosphere communities. Mean and standard deviation values of inoculated plants under herbivory and no-herbivory stress. Alpha diversity indexes Chao 1, Inverse Simpson and Observed were tested for differences between treatments with one-way ANOVA. Within each stress condition, post-hoc Tukey HSD tests confirmed significant differences (p<0.05) between treatments (groups) are indicated with different letters.

|  | **Chao 1** |  | | | **Inv. Simpson** |  | | | **Observed** |  | |  |
| --- | --- | --- | --- | --- | --- | --- | --- | --- | --- | --- | --- | --- |
| **Treatment** | Mean ± sd | | grp | Mean ± sd | | | grp | Mean ± sd | | | grp | |
| *No herbivory* | | | | | | | | | | | | |
| Control | 202.97 ± 20.05 | | a | 15.82 ± 1.82 | | | bc | 191.17 ± 10.91 | | | a | |
| Ba | 182.07 ± 38.36 | | a | 13.83 ± 8.87 | | | bc | 173.4 ± 37.09 | | | a | |
| Pa | 179.57 ± 33.71 | | a | 12.59 ± 6.88 | | | c | 173.29 ± 32.49 | | | a | |
| Th | 186.81 ± 19.66 | | a | 20.38 ± 4.1 | | | ab | 182.33 ± 17.42 | | | a | |
| Ri | 204.1 ± 19.55 | | a | 18.32 ± 6.09 | | | ab | 198.83 ± 18.04 | | | a | |
| SynCom | 201.75 ± 15.22 | | a | 22.38 ± 2.24 | | | a | 198.17 ± 12.73 | | | a | |
| *Herbivory* |  | |  |  | | |  |  | | |  | |
| Control | 200.64 ± 19.11 | | a | 21.3 ± 2.65 | | | ab | 197.17 ± 18.79 | | | a | |
| Ba | 222.18 ± 29.36 | | a | 17.82 ± 1.28 | | | bc | 213.5 ± 28.6 | | | a | |
| Pa | 202.18 ± 28.28 | | a | 18.85 ± 7.51 | | | ab | 196.67 ± 25.07 | | | a | |
| Th | 192.52 ± 28.72 | | a | 22.99 ± 2.29 | | | a | 185.67 ± 25.89 | | | a | |
| Ri | 197.63 ± 42.79 | | a | 19.06 ± 6.6 | | | ab | 188.5 ± 37.24 | | | a | |
| SynCom | 210.97 ± 13.39 | | a | 18.61 ± 6.85 | | | ab | 205.67 ± 12.19 | | | a | |

**Figure S5.** ASVs differential analysis of fungal communities vs Control under non-herbivory (H-).

**
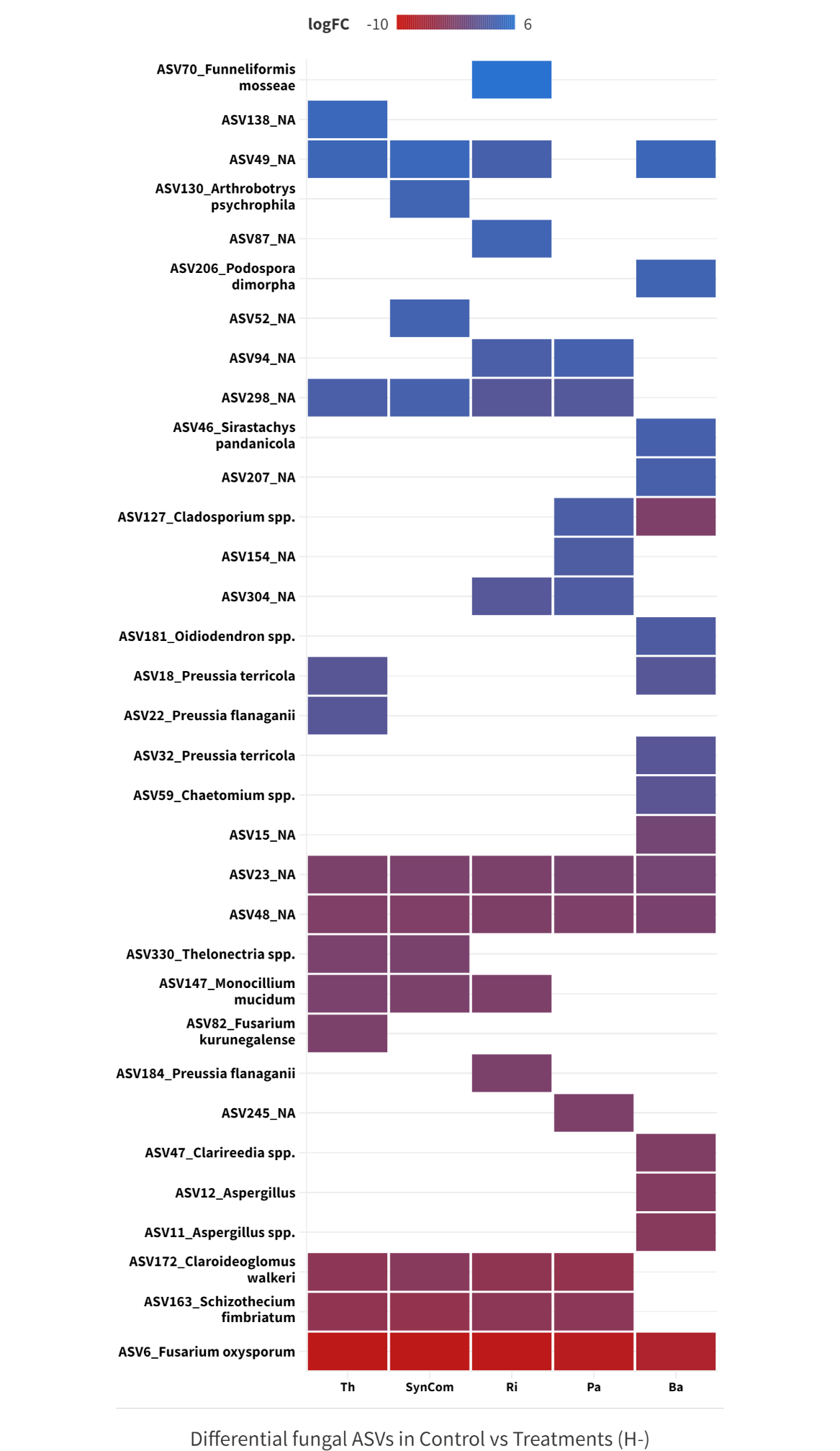
**

**Figure S6.** ASVs differential analysis of fungal communities vs Control under herbivory (H+).

**
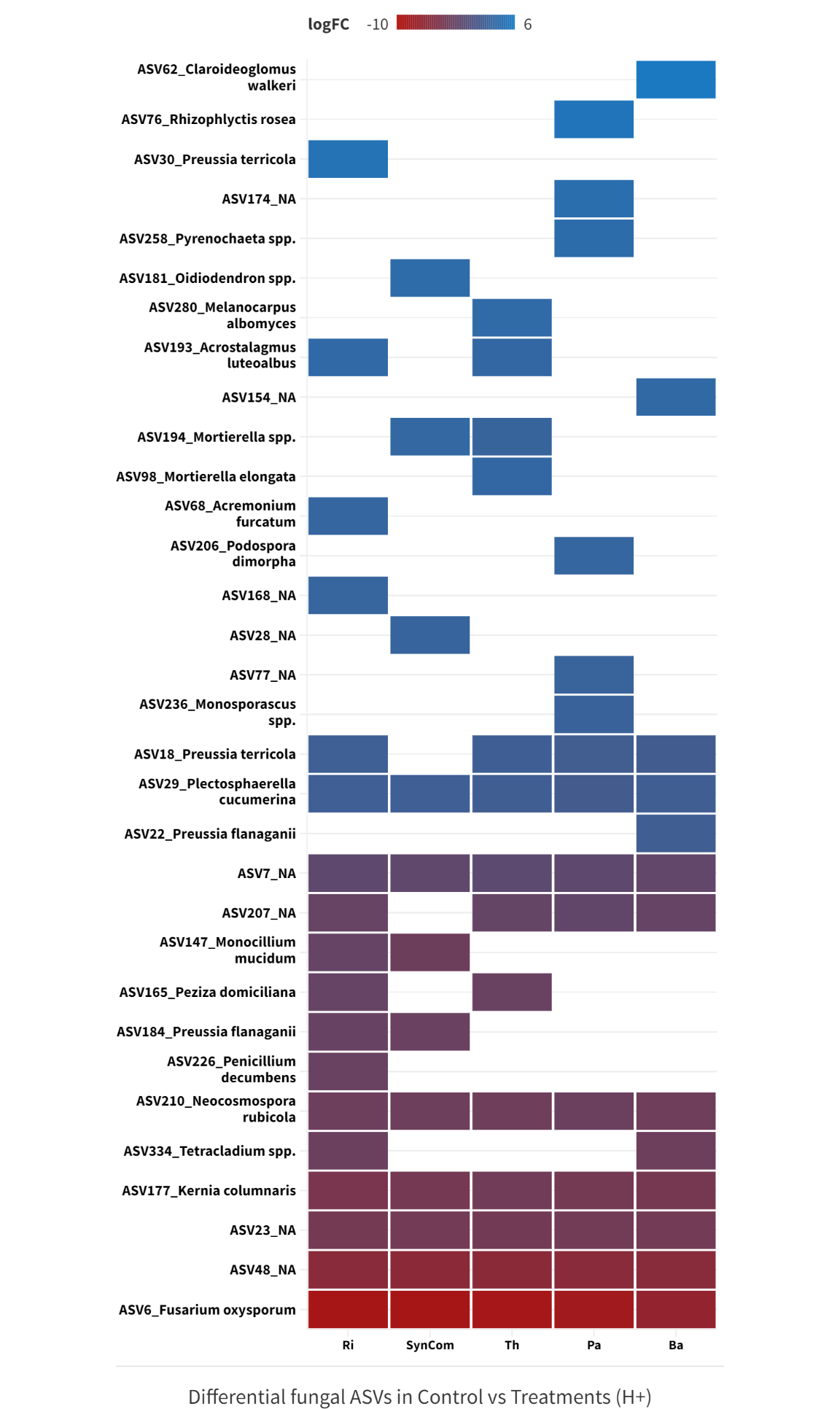
**

**Table S8.** Rhizosphere volatile compounds of tomato plants before leaf herbivory (T0) and 24 h after leaf herbivory stress (T1). Mean peak intensity of six replicates per microbial inoculant treatment; Control (non-inoculated), Ba (*B. amyloliquefaciens*), Pa (*P.azotoformans*), Th (*T. harzianum*), Ri (*R. irregularis*) and synthetic community (SynCom). Colour gradient for each compound across microbial treatments and herbivory conditions from lower (blue) to higher (red) peak intensity.

|  | **Non-herbivory (T0)** | | | | | | **Herbivory (T1)** | | | | | |
| --- | --- | --- | --- | --- | --- | --- | --- | --- | --- | --- | --- | --- |
| **Compound** | Control | Ba | Pa | Th | Ri | SynCom | Control | Ba | Pa | Th | Ri | SynCom |
| acetic acid | 5.30E+04 | 0.00E+00 | 3.37E+04 | 1.08E+05 | 7.04E+04 | 3.27E+04 | 5.70E+04 | 4.31E+04 | 3.33E+04 | 4.29E+04 | 4.22E+04 | 1.95E+04 |
| dimethyl disulfide | 6.92E+04 | 1.38E+05 | 2.62E+04 | 1.79E+05 | 9.94E+04 | 8.70E+05 | 2.32E+06 | 1.18E+06 | 6.43E+05 | 1.97E+05 | 6.21E+05 | 1.41E+06 |
| octane | 4.75E+03 | 0.00E+00 | 0.00E+00 | 9.43E+03 | 3.32E+03 | 2.63E+04 | 8.86E+03 | 1.59E+04 | 0.00E+00 | 1.48E+04 | 2.68E+04 | 8.98E+03 |
| hexanal | 2.39E+04 | 1.83E+04 | 1.95E+04 | 1.81E+04 | 2.97E+04 | 1.19E+04 | 3.44E+04 | 2.86E+04 | 2.92E+04 | 6.13E+04 | 6.84E+04 | 3.12E+04 |
| nonane | 0.00E+00 | 0.00E+00 | 1.43E+03 | 2.81E+03 | 0.00E+00 | 1.67E+03 | 4.07E+03 | 2.34E+03 | 0.00E+00 | 6.21E+03 | 3.10E+03 | 4.20E+03 |
| unknown 954 | 2.44E+04 | 3.35E+04 | 2.73E+04 | 1.64E+04 | 4.59E+03 | 1.58E+04 | 1.57E+04 | 1.71E+04 | 1.96E+04 | 1.95E+04 | 1.17E+04 | 1.90E+04 |
| benzaldehyde | 1.42E+06 | 1.46E+06 | 1.38E+06 | 1.45E+06 | 1.12E+06 | 3.02E+06 | 1.21E+06 | 1.10E+06 | 7.63E+05 | 1.31E+06 | 2.05E+06 | 1.36E+06 |
| dimethyl trisulfide | 1.65E+03 | 7.90E+03 | 0.00E+00 | 1.14E+04 | 5.71E+03 | 9.96E+04 | 1.29E+05 | 5.13E+04 | 3.42E+04 | 6.95E+03 | 4.40E+04 | 8.29E+04 |
| unknown 985 | 0.00E+00 | 5.32E+03 | 0.00E+00 | 1.64E+04 | 3.64E+04 | 9.11E+03 | 1.27E+04 | 2.07E+04 | 1.51E+04 | 1.34E+04 | 2.90E+04 | 1.05E+04 |
| benzonitrile | 2.19E+05 | 2.54E+05 | 1.77E+05 | 2.28E+05 | 1.47E+05 | 2.00E+06 | 1.53E+05 | 1.27E+05 | 1.12E+05 | 1.67E+05 | 3.65E+05 | 1.93E+05 |
| 2-pentyl furan | 8.83E+03 | 2.71E+03 | 2.77E+03 | 1.76E+04 | 6.03E+03 | 8.81E+03 | 6.14E+03 | 8.58E+03 | 3.43E+03 | 4.42E+04 | 4.97E+04 | 1.46E+04 |
| benzofuran | 2.33E+05 | 3.14E+04 | 2.36E+04 | 1.58E+04 | 6.32E+03 | 5.78E+04 | 7.80E+04 | 1.08E+05 | 1.65E+05 | 0.00E+00 | 1.91E+05 | 3.30E+04 |
| octanal | 2.72E+04 | 2.14E+04 | 2.20E+04 | 4.84E+04 | 4.83E+04 | 4.51E+04 | 4.85E+04 | 6.09E+04 | 4.05E+04 | 7.56E+04 | 6.68E+04 | 4.83E+04 |
| p-cymene | 2.85E+06 | 2.84E+05 | 5.92E+04 | 1.72E+05 | 1.00E+05 | 7.24E+04 | 9.13E+04 | 2.09E+06 | 1.97E+05 | 2.44E+06 | 2.62E+05 | 1.08E+06 |
| 2-ethyl-1-hexanol | 9.80E+04 | 1.25E+05 | 9.38E+04 | 1.79E+05 | 1.36E+05 | 1.39E+05 | 1.61E+05 | 1.58E+05 | 1.16E+05 | 1.79E+05 | 2.46E+05 | 1.87E+05 |
| limonene | 0.00E+00 | 2.74E+04 | 0.00E+00 | 1.68E+04 | 1.49E+04 | 5.93E+03 | 6.08E+03 | 5.26E+05 | 1.28E+04 | 8.44E+05 | 8.06E+04 | 2.40E+05 |
| benzyl alcohol | 0.00E+00 | 1.48E+04 | 0.00E+00 | 3.91E+04 | 1.37E+04 | 7.64E+03 | - | - | - | - | - | - |
| β-phellandrene | 0.00E+00 | 3.86E+04 | 0.00E+00 | 1.16E+05 | 2.29E+04 | 1.78E+04 | - | - | - | - | - | - |
| benzene acetaldehyde | 0.00E+00 | 5.23E+03 | 1.19E+04 | 5.76E+04 | 4.45E+04 | 7.72E+04 | 6.08E+03 | 5.26E+05 | 1.28E+04 | 8.44E+05 | 8.06E+04 | 2.40E+05 |
| acetophenone | 7.81E+05 | 8.04E+05 | 8.50E+05 | 1.03E+06 | 5.38E+05 | 2.23E+06 | 4.81E+04 | 4.22E+04 | 2.57E+04 | 4.38E+04 | 5.62E+04 | 3.52E+04 |
| nonanal | 1.85E+04 | 3.02E+04 | 5.62E+04 | 8.24E+04 | 1.02E+05 | 9.89E+04 | 4.76E+05 | 4.12E+05 | 3.25E+05 | 4.62E+05 | 6.20E+05 | 5.51E+05 |
| unknown1190 | 2.02E+04 | 4.51E+03 | 0.00E+00 | 1.16E+04 | 6.38E+03 | 0.00E+00 | 7.03E+04 | 9.93E+04 | 6.67E+04 | 9.63E+04 | 7.70E+04 | 7.54E+04 |
| decanal | 3.57E+04 | 6.79E+04 | 1.59E+05 | 1.63E+05 | 1.53E+05 | 1.16E+05 | 0.00E+00 | 1.44E+04 | 0.00E+00 | 3.07E+04 | 6.52E+03 | 3.00E+03 |
| benzothiazole | 6.27E+03 | 3.71E+03 | 4.56E+03 | 0.00E+00 | 0.00E+00 | 3.52E+03 | 1.04E+05 | 2.05E+05 | 7.19E+04 | 1.16E+05 | 7.50E+04 | 1.07E+05 |
